# Single-Electron Redox Chemistry on the [Cp*Rh] Platform Enabled by a Nitrated Bipyridyl Ligand

**DOI:** 10.3390/molecules23112857

**Published:** 2018-11-02

**Authors:** William N. G. Moore, Wade C. Henke, Davide Lionetti, Victor W. Day, James D. Blakemore

**Affiliations:** Department of Chemistry, University of Kansas, 1251 Wescoe Hall Drive, Lawrence, KS 66045, USA; will.n.g.moore@ku.edu (W.N.G.M.); whenke@ku.edu (W.C.H.); dlionett@ku.edu (D.L.); vwday@ku.edu (V.W.D.)

**Keywords:** rhodium, electrochemistry, paramagnetic, spectroelectrochemistry, catalysis

## Abstract

[Cp*Rh] complexes (Cp* = pentamethylcyclopentadienyl) are attracting renewed interest in coordination chemistry and catalysis, but these useful compounds often undergo net two-electron redox cycling that precludes observation of individual one-electron reduction events. Here, we show that a [Cp*Rh] complex bearing the 4,4′-dinitro-2,2′-bipyridyl ligand (dnbpy) (**3**) can access a distinctive manifold of five oxidation states in organic electrolytes, contrasting with prior work that found no accessible reductions in aqueous electrolyte. These states are readily generated from a newly isolated and fully characterized rhodium(III) precursor complex **3**, formulated as [Cp*Rh(dnbpy)Cl]PF_6_. Single-crystal X-ray diffraction (XRD) data, previously unavailable for the dnbpy ligand bound to the [Cp*Rh] platform, confirm the presence of both [η^5^-Cp*] and [κ^2^-dnbpy]. Four individual one-electron reductions of **3** are observed, contrasting sharply with the single two-electron reductions of other [Cp*Rh] complexes. Chemical preparation and the study of the singly reduced species with electronic absorption and electron paramagnetic resonance spectroscopies indicate that the first reduction is predominantly centered on the dnbpy ligand. Comparative cyclic voltammetry studies with [NBu_4_][PF_6_] and [NBu_4_][Cl] as supporting electrolytes indicate that the chloride ligand can be lost from **3** by ligand exchange upon reduction. Spectroelectrochemical studies with ultraviolet (UV)-visible detection reveal isosbestic behavior, confirming the clean interconversion of the reduced forms of **3** inferred from the voltammetry with [NBu_4_][PF_6_] as supporting electrolyte. Electrochemical reduction in the presence of triethylammonium results in an irreversible response, but does not give rise to catalytic H_2_ evolution, contrasting with the reactivity patterns observed in [Cp*Rh] complexes bearing bipyridyl ligands with less electron-withdrawing substituents.

## 1. Introduction

The development of metal complexes capable of efficiently storing and transferring reducing equivalents attracts interest in a variety of contexts. Reduced metal complexes are key reaction intermediates in many transformations, including photoredox reactions enabled by reductive quenching pathways [1], olefin polymerization or oligomerization [2], and reactions involving concerted oxidative addition to transition metal complexes [3]. Reduced metal complexes also attract significant attention in the area of molecular electrocatalysis [4], as several reduced intermediates are typically involved in multielectron redox cycles that generate fuels like H_2_ via H^+^ and e^−^ coupling [5,6]. In many of these cycles, however, the key metal complexes reduced by one or more e^−^ equivalents are not isolated or detected—instead, their involvement is inferred from the observed reactivity [7].

**Scheme 1:**
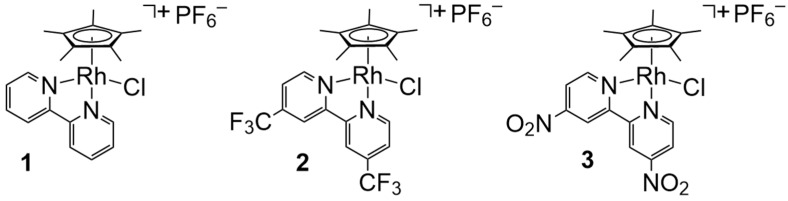
[Cp*Rh] complexes discussed in this study.

[Cp*Rh] complexes (Cp* = pentamethylcyclopentadienyl, Scheme 1) are one class of catalysts for H^+^ and e^−^ coupling that are capable of generating H_2_ from water [8]. These catalysts are supported by bidentate chelating ligands, such as 2,2′-bipyridyl (bpy, as in complex **1**) and its substituted derivatives [8,9,10]. [Cp*] and diimine ligands (like bpy) are readily installed through straightforward synthetic chemistry onto the rhodium center in these compounds [10,11], and thus this system has been popular for model studies of H_2_ generation [12,13] as well as applications in other areas [14]. In this work, two-electron reduction of the rhodium(III) precatalyst in the presence of a suitably strong acid results in quantitative formation of H_2_ and regeneration of the starting rhodium(III) complexes [10]. However, in chemistry that is distinctive for this family of catalysts, the putative rhodium(II) intermediate generated by the initial 1e^−^ reduction of a given precatalyst does not result in a stable intermediate. In chemical work, treatment of Rh^III^ with 1 equiv. of reducing agent results in disproportionation of two transient Rh^II^ complexes to yield 0.5 equiv. each of Rh^I^ and Rh^III^ [9]. In electrochemical work, a so-called ECE-type event occurs at the electrode: a single reductive wave is observed by cyclic voltammetry, corresponding to the first reduction of Rh^III^ (E), followed by a fast chemical reaction step (C), and then immediate transfer of a second electron (E′) to reduce Rh^II^ to Rh^I^ [15]. Thus, Rh^I^ is very quickly generated following formation of Rh^II^, because the *E*(Rh^II^/Rh^I^) is more positive than *E*(Rh^III^/Rh^II^). These two-electron events obscure the routine measurement of the individual one-electron reduction events involved in this chemistry [7,10,16].

The energy conversion efficiency of these [Cp*Rh] catalysts is closely tied to the potentials of the individual reduction events [7,17,18], but only a limited level of control of these parameters has been demonstrated to date. Moreover, the use of redox-active bpy ligands (and other diimines) in these complexes has attracted significant attention [19,20,21]. Understanding the nature of the observed reductions is key, as these [Cp*Rh] complexes bearing diimine ligands undergo unique [Cp*]-centered protonations [22,23] during catalysis to generate [Cp*H] complexes [24] that are active for H_2_ evolution. Our group recently showed that installation of electron-withdrawing trifluoromethyl groups at the 4 and 4′ positions of the bpy ligand (as in **2**) results in a previously unobserved catalytic pathway involving reduction of the 4,4′-bis(trifluoromethyl)-2,2′-bipyridyl ligand on [(Cp*H)Rh^I^] species, followed by H_2_ evolution [10]. We have also found that [Cp*Rh] complexes bearing bidentate diphosphine [25] or hybrid phosphine-quinoline ligands [26] are not capable of similar catalysis. Thus, although the role of supporting bidentate ligand structure in formation of [Cp*H] intermediates is not yet clear, it is of high interest considering the new reactivity manifolds that may be accessible with [Cp*H] complexes [27,28].

As use of 4,4′-bis(trifluoromethyl)-2,2′-bipyridyl enables observation of a new catalytic pathway with **2** [10], we became interested in the electrochemical behavior engendered by use of the 4,4′-dinitro-2,2′-bipyridyl (dnbpy) ligand, which by contrast features electron-withdrawing nitro groups (–NO_2_). In considering use of the dnbpy ligand, it is useful to note the Hammett parameter (σ^−^) values associated with the hydrogen (–H), trifluoromethyl (–CF_3_), and nitro functionalities (–NO_2_). Specifically, these values are 0, 0.65, and 1.27, respectively [29]. Thus, in terms of the effects engendered by substituents at the 4 and 4′ positions on electronic properties, moving from –CF_3_ to –NO_2_ represents a similarly significant difference as moving from –H to –CF_3_.

We were pleased to find that Lütz and co-workers previously reported preparation of [Cp*Rh(dnbpy)Cl]Cl [30], and that [Cp*Ir] complexes bearing dnbpy have been known for some years [31,32,33]. Moreover, dnbpy [34,35,36] and other nitrated polypyridyls [37,38] have been explored as ligands to rhodium in other frameworks. We were especially intrigued to read that Lütz and co-workers found that [Cp*Rh(dnbpy)Cl]Cl did not undergo electrochemical reduction down to –1 V vs. Ag/AgCl in aqueous electrolyte [30]. However, in their report, [Cp*Rh(dnbpy)Cl]Cl was prepared for use in a high-throughput, robotic electrochemical system, rather than fully characterized with proof of homogeneity and bulk composition from synthetic work [30]. Moreover, the electrochemical studies were carried out in aqueous electrolyte, rather than the organic electrolytes common in studies involving organometallic compounds.

Here, we now report the isolation, full characterization, and electrochemical studies of [Cp*Rh(dnbpy)Cl]PF_6_ (**3**). We find that use of dnbpy engenders unique electrochemical properties in organic solvent-based electrolytes, as **3** undergoes three quasi-reversible one-electron reduction events and an additional, one-electron reduction event that is irreversible. The first, one-electron reduced product of **3** can be generated chemically and isolated, and spectroscopic work confirms that the first electron transferred is stored in orbitals primarily associated with the dnbpy ligand. Spectroelectrochemical studies reveal the clean interconversion of **3** and its reduction products, as isosbestic behavior is observed during multistep polarization experiments. The addition of acid in the form of triethylammonium bromide (p*K*_a_ ≈ 19 in MeCN [39]) does not result in generation of diamagnetic [Cp*H] complexes or hydrides, and does not lead to catalytic activity toward H_2_ evolution. These results are discussed in the context of understanding and guiding the order and energetics of e^−^ and H^+^ delivery to complexes assembled with the [Cp*Rh] fragment. 

## 2. Results

In order to study the properties of dnbpy complexes containing the [Cp*Rh] fragment, we targeted synthesis of [Cp*Rh(dnbpy)Cl]PF_6_ (**3**). We have encountered cleaner reactivity of the [Cp*RhCl_2_]_2_ precursor [11] upon use of silver reagents to remove one equivalent of chloride from each rhodium center, motivating preparation of the hexafluorophosphate salt **3** [40,41]. We first synthesized dnbpy with literature methods, routinely obtaining an overall yield of ca. 50% [42,43,44,45,46,47]. **3** was then prepared in tetrahydrofuran (THF) by addition of 2 equiv. of dnbpy to [Cp*RhCl_2_]_2_, followed by addition of 2 equiv. of AgPF_6_, resulting in formation of the rhodium(III) complex **3** in moderate 44% yield (See Experimental Section and SI, Figures S1–S4, S6, S7 for characterization data).

**Figure 1:**
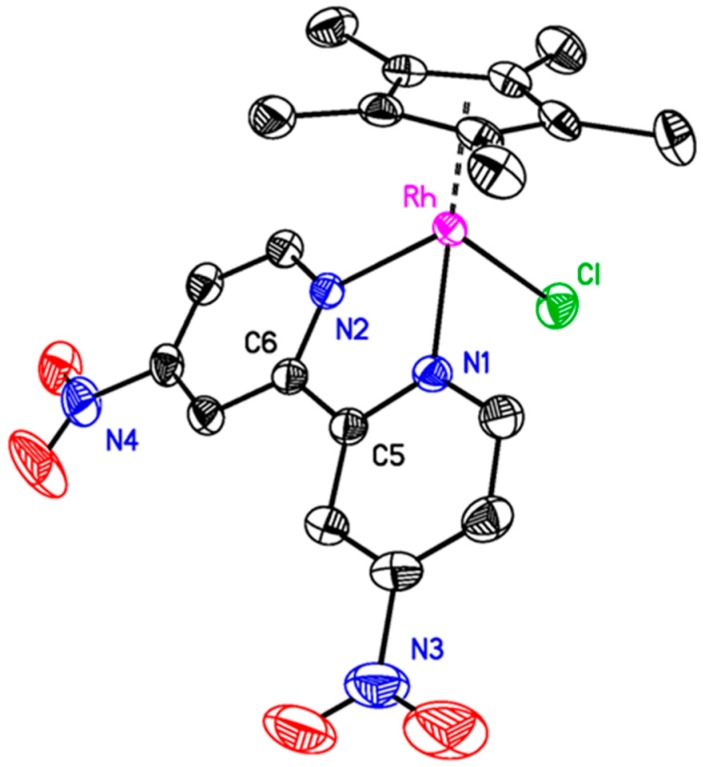
Solid-state structure (X-ray diffraction (XRD)) of **3**. H atoms, PF_6_^−^ counteranion, and one co-crystallized MeCN molecule omitted for clarity. Displacement ellipsoids are shown at 50% probability.

Vapor diffusion of diethyl ether into a concentrated acetonitrile (MeCN) solution of **3** yielded orange crystals suitable for single-crystal X-ray diffraction (XRD) studies. The geometry at the rhodium center is pseudo-octahedral, with a first coordination sphere around the metal center containing [η^5^-Cp*], [κ^2^-dnbpy], and a bound chloride anion (see Figure 1). The geometry and metal-ligand distances do not differ significantly from other structures of [Cp*Rh^III^] complexes containing 4,4′-disubstituted-2,2′-bipyridyl ligands [10]. However, only a limited number of XRD datasets are available in the Cambridge Structural Database for metal complexes of dnbpy, and our structure of **3** is the first structure obtained with rhodium [48]. In the structure of **3**, as in most other structures containing dnbpy, the (NO_2_) groups are approximately co-planar with their partnered pyridine rings. In fact, of the seven total structures of dnbpy itself [49], or those containing dnbpy [50,51,52,53,54,55], only one of these [51] has an O–N–C–C torsion angle greater than 13°. The observed co-planarity of the NO_2_ groups and the pyridine rings suggests that there is likely strong electronic communication between these substituents and the π system of bipyridine. Therefore, we turned to electrochemical methods to establish the influence of the nitro groups on the electrochemical properties of the metal complex.

**Figure 2:**
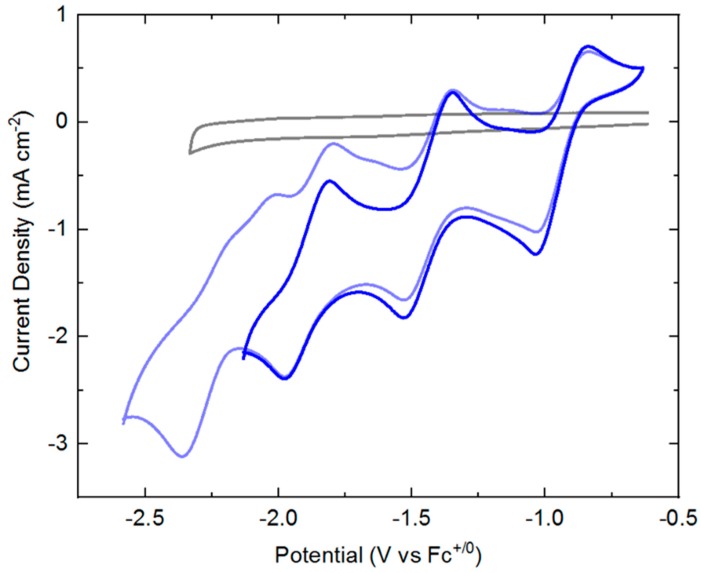
Cyclic voltammograms of **3**. Scan rate: 100 mV/s. Dark blue line: Cathodic sweep from ca. −0.6 V to a switching potential of −2.2 V and returning to −0.6 V. Light blue line: Cathodic sweep from ca. −0.6 V to a switching potential of −2.6 V and returning to −0.6 V. Gray line: electrolyte-only blank. Conditions: [**3**] ≈ 1 × 10^−3^ M; electrolyte: 0.1 M [NBu_4_][PF_6_] in tetrahydrofuran (THF).

Cyclic voltammograms (CVs) of **3** (ca. 1 mM) were collected in THF solution containing 0.1 M tetrabutylammonium hexafluorophosphate ([NBu_4_][PF_6_]) as supporting electrolyte. Beginning at oxidizing potentials, **3** displays a manifold of four reduction events (see Figure 2) that onset around −1 V versus the ferrocenium/ferrocene couple (denoted hereafter as Fc^+/0^). The key parameters associated with each of these four reduction events are summarized in Table 1. If the switching potential for the return anodic sweep is set at −2.2 V, the first three reduction events appear to be quasi-reversible with well-defined, clean return anodic waves. However, if the switching potential is set at a greater negative value of −2.6 V, the fourth reduction wave is clearly visible. This fourth reduction, however, is not accompanied by a clean return oxidation wave, suggesting that one or more significant chemical reactions may follow injection of a fourth electron into the rhodium complex. Interrogation of the scan rate dependence of the both the cathodic and anodic peak currents for the first three observed redox processes reveals a square-root dependence (see SI, Figures S13–S15). This indicates that all the oxidized and reduced forms of the complex undergoing reduction and oxidation are freely diffusional in solution and homogeneous.

We also carried out cyclic voltammetry of **3** in acetonitrile electrolyte, and consistently observed a similar manifold of reduction events (see SI, Figure S10). Specifically, three quasi-reversible reductions are followed by a virtually irreversible reduction, albeit at shifted potentials (see [56] for further discussion). However, we conducted most of our studies in THF electrolyte, as the complex typically yielded a better response under these conditions.

In the cyclic voltammetry shown in Figure 2, the peak heights of the reduction processes appear to be similar, suggesting that the same number of electrons are transferred during each event. However, as the individual reduction events are relatively closely spaced, estimation of the appropriate background-corrected peak heights could be challenging. Therefore, differential pulse voltammetry (DPV) was carried out to quantitatively examine the number of electrons transferred in each event. For this determination, we prepared a solution containing a 1:1 mixture of ferrocene (Cp_2_Fe) and **3** in THF containing 0.1 M [NBu_4_][PF_6_] supporting electrolyte and collected a differential pulse voltammogram from +0.5 V to −2.6 V (see SI, Figure S12). In addition to the one-electron process corresponding to the Fe^III^/Fe^II^ couple of ferrocene, we observe four closely spaced and reasonably well resolved processes over a range similar to that seen for **3** in cyclic voltammetry. The areas of the four processes measured for **3** and that of Cp_2_Fe were fit to Gaussian profiles, and comparison of the peak areas to that of the internal ferrocene standard confirms that one electron is indeed transferred in each event (see SI, Table S3 for peak area ratios).

The electrochemical response of complex **3** sharply contrasts with the behavior commonly encountered for other [Cp*Rh^III^] complexes. Most other chloride-bound complexes in this family containing other diimine [8,10,24,40], diphosphine [9,25], or hybrid phosphine-monoimine ligands [26] undergo a net two-electron reduction that appears as a single redox process in cyclic voltammetry experiments. As described in the Introduction, this ECE-type electrochemical response implicates that a chemical reaction follows the initial reduction of the metal complex and leads to formation of a species that undergoes immediate transfer of a second electron [7,15]. Disentangling the nature of the elementary steps in this chemistry is of high interest, as the resulting 2e^−^-reduced complexes often undergo subsequent reactivity with protons. Notably, our recent work examining the case of a [Cp*Rh] complex bearing the hybrid 8-(diphenylphosphino)quinoline (PQN) ligand suggests that the first reduction of [Cp*Rh^III^(PQN)Cl]PF_6_ is rhodium-centered, and leads to ejection of the chloride ligand at the Rh^II^ oxidation state [26]. The electrochemical behavior of the [Cp*Rh] complex supported by the dimethyldipyridylmethane (Me_2_dpma) ligand is also consistent with initial metal-centered reduction [40].

Therefore, to investigate the nature of the first reduction of **3**, we targeted preparation of the singly reduced product. In accord with the clean, one-electron reduction of **3** observed by cyclic voltammetry, treatment of a THF suspension of **3** with cobaltocene (*E*° ≈ −1.3 V, [57] 2 equiv.) results in an immediate color change from bright yellow to a deep shade of forest green. Following stirring for 10 min and the subsequent removal of all volatiles under vacuum, the reduction product **4** was extracted with THF and isolated as a dark green solid. Characterization of **4** by ^1^H-nuclear magnetic resonance (NMR) (Figure S5) reveals a loss of all resonances associated with **3**, including those of the [κ^2^-dnbpy] ligand in the aromatic region and that associated with [η^5^-Cp*] in the aliphatic region. The disappearance of these resonances and lack of new peaks associated with diamagnetic material is consistent with generation of a paramagnetic complex, as would be expected for a 1e^−^ reduction of **3** as observed in cyclic voltammetry. A small impurity of [Cp_2_Co]^+^ (δ = 5.66 ppm) is observed in NMR spectra of isolated samples from the reduction of **3**, but could not be removed due to the similar solubility profiles of **4** and cobaltocenium.

**Figure 3:**
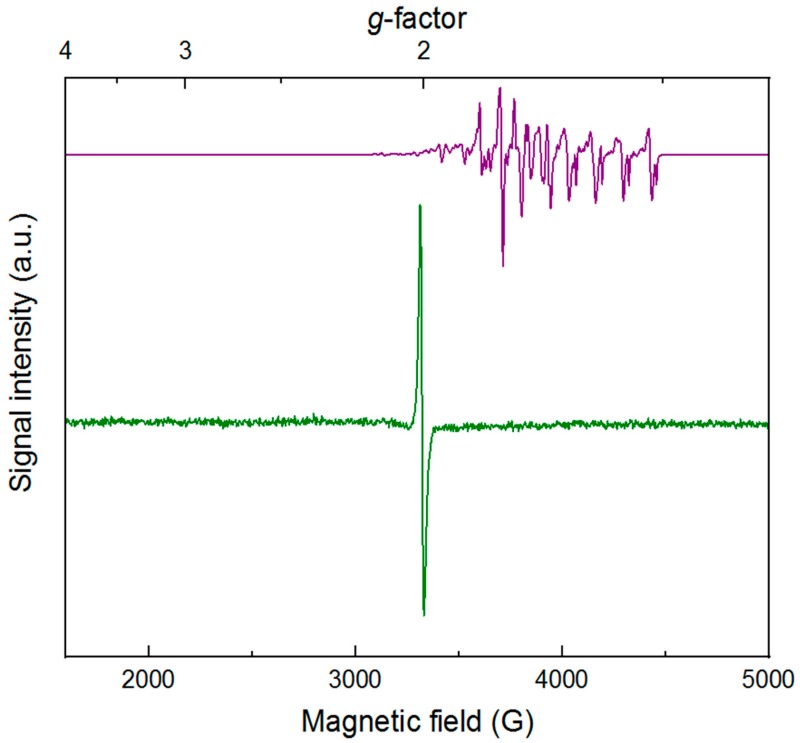
X-band CW EPR spectrum of **4** (green line) and cobaltocene (purple line). Conditions: *T* = 10 K; modulation amplitude = 2.0 G; time constant = 20.5 ms; [**4**] = 10^−3^ M.

To further characterize **4**, we turned to electron paramagnetic resonance spectroscopy. Prior to reduction, **3** is a low-spin rhodium(III) complex with a *d*^6^ configuration and *S* = 0. The cobalt(II) reductant (Cp_2_Co) used to generate **4** has a *d*^7^ configuration and is a *S* = 1/2 species, displaying the distinctive spectrum (consistent with literature) shown as the purple line in Figure 3. This spectrum displays hyperfine coupling to the *I* = 7/2 cobalt nucleus. In contrast, the spectrum of **4** isolated as described above reveals a relatively narrow and isotropic signal with a center crossing point at *g* = 2.006 (*H* = 3341 G). Although **4** could be considered to be a formal rhodium(II) species, the sharp and isotropic spectrum is instead consistent with an organic radical—in this case, predominant localization of unpaired electron density on the dnbpy ligand. Thus, **4** can be most appropriately considered as a rhodium(III) complex with a bound dnbpy^∙−^. Retention of this ligand radical in the first coordination sphere of the rhodium center is consistent with both the quasi-reversible CV studies (vide supra) and spectroelectrochemical work that confirms the chemically reversible interconversion of **3** and **4** (vide infra) on the seconds to minutes timescale. Moreover, the lack of resolved hyperfine coupling to the *I* = ½ ^103^Rh nucleus (100% abundance) in **4** corroborates assignment of the reduced metal species as having unpaired electron density that is localized primarily on dnbpy. Notably, the trace impurity of [Cp_2_Co]^+^ present in samples of isolated **4** does not contribute to the EPR spectrum shown in Figure 3, as [Cp_2_Co]^+^ is an *S* = 0 low-spin cobalt(III) complex.

Few examples of formal rhodium(II) complexes have been observed by EPR [58]. Localization of unpaired electron density in orbitals with increased Rh(II) character would be expected to result in a significantly more anisotropic spectrum than that observed for **4**, with larger *g*-value shifts and resolved hyperfine coupling to the metal nucleus. The experimental data for **4** compare well with data that we have recently obtained on [Cp*Rh(bpy^∙−^)Me]^0^ and [Cp*Ir(bpy^∙−^)Me]^0^ compounds [41]. Specifically, these methyl complexes display narrow rhombic spectra centered near *g* ≈ 2.0. This greater rhombicity arises from hyperfine couplings to the *I* = 1/2 Rh^III^ and *I* = 3/2 Ir^III^ centers in these compounds, contrasting with the case of virtually ligand-centered **4**. Thus, we conclude that the unpaired electron density on dnbpy^∙−^ is contained in molecular orbitals with very little character arising from rhodium, a phenomenon likely driven by the presence of the strongly electron-withdrawing nitro groups on dnbpy.

**Figure 4:**
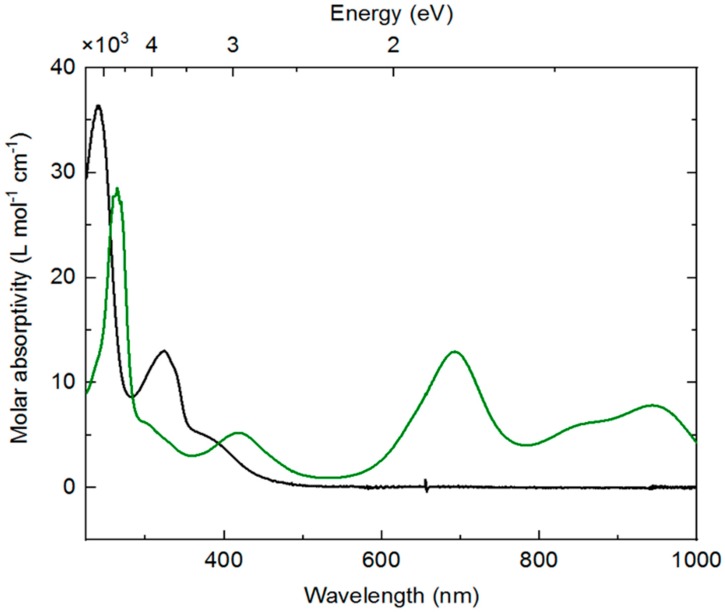
Electronic absorption spectra of **3** (black line) and **4** (green line). Conditions: [**3**] ≈ [**4**] ≈ 10^−5^; solvent: tetrahydrofuran.

To gain further insight into the dnbpy-localization of electron density arising from the first reduction of **3**, we turned to electronic absorption spectroscopy (see Figure 4 and SI, Figure S8). The spectrum of **3** is unremarkable and displays features consistent with most rhodium(III) complexes; **3** is a yellow solid, and the ultraviolet (UV)-visible absorption spectrum reflects this with a relatively intense (ca. 5000 M^−1^ cm^−1^) band trailing into the visible around 400 nm. Isolated **4** displays a very different profile, with distinctive new features in the visible-near infrared (NIR) region (λ_max_ values at 694 nm, 860 nm, and 945 nm with molar absorptivities of 13,000, 6100, and 7800 M^−1^ cm^−1^, respectively). Consistent with the observed forest-green color of **4**, a weaker absorption band is retained at lower wavelength (420 nm, 5200 M^−1^ cm^−1^) and thus transmits predominantly green light between these bands. The absorption bands in the 800–1000 nm range are similar to examples of both free bpy^∙−^ and metal complexes ligated by [bpy^∙−^] [59,60,61]. Analogous data is not available from prior work for dnbpy^∙−^, although similar features are measured for the doubly reduced form of complex **2**, which possesses significant reduced-ligand character [21]. Thus, both EPR and electronic absorption data are consistent with assignment of dnbpy-centered reduction in **4**.

Metal-centered reduction of [Cp*Rh^III^] complexes is associated with ejection of monodentate ligands such as chloride from the first coordination sphere. This phenomenon is driven by formation of transient, 19e^−^ Rh^II^ intermediates upon reduction that release the monodentate ligand to form more stable 17e^−^ species [26]. The assignment of ligand-centered reduction of **3** thus prompted us to explore whether analogous reactivity takes place here. To test this, we performed cyclic voltammetry on **3** in acetonitrile containing 0.1 M [NBu_4_][Cl] as supporting electrolyte. The first reduction of **3** remains unchanged from the case of [NBu_4_][PF_6_], showing a quasi-reversible appearance. However, the appearance of the second reduction of **3** (or reduction of **4**) is different, showing two peaks on the cathodic sweep (Δ*E* ≈ 175 mV) and a single peak on the anodic sweep (see SI, Figure S11). This is consistent with the formation of both the cationic solvento complex [Cp*Rh(dnbpy^∙−^)(NCMe)](PF_6_) and a neutral chloride complex Cp*Rh(dnbpy^∙−^)Cl following the first reduction, when the electrolyte contains a 100-fold excess of chloride. Thus, under conditions where chloride is not found in excess (Figure 2), we propose that reduction of **3** leads exclusively to generation of the 18e^−^, cationic [Cp*Rh(dnbpy^∙−^)(NCMe)](PF_6_) complex. Consistent with this significant coupled chemical reaction, the peak-to-peak separation (Δ*E*_p_ ≈ 200 mV in THF, 90 mV in MeCN) associated with reduction of **3** to **4** in [NBu_4_][PF_6_] is significantly larger than those associated with the following two reductions (180, 170 mV in THF; 70, 70 mV in MeCN) (see Table 1 and Table S1 in SI). Completing our proposed model for the electrochemistry, reduction of **4** leads to a single product, on the basis of the single anodic wave observed at −1.35 V in THF (Figure 2) and −1.19 V in MeCN (see SI, Figure S10). As this species is rather electron-rich, we speculate that the reduction of **4** produces [Cp*Rh(dnbpy)]^0^; however, further assignments regarding this compound are beyond the scope of this study. 

**Figure 5:**
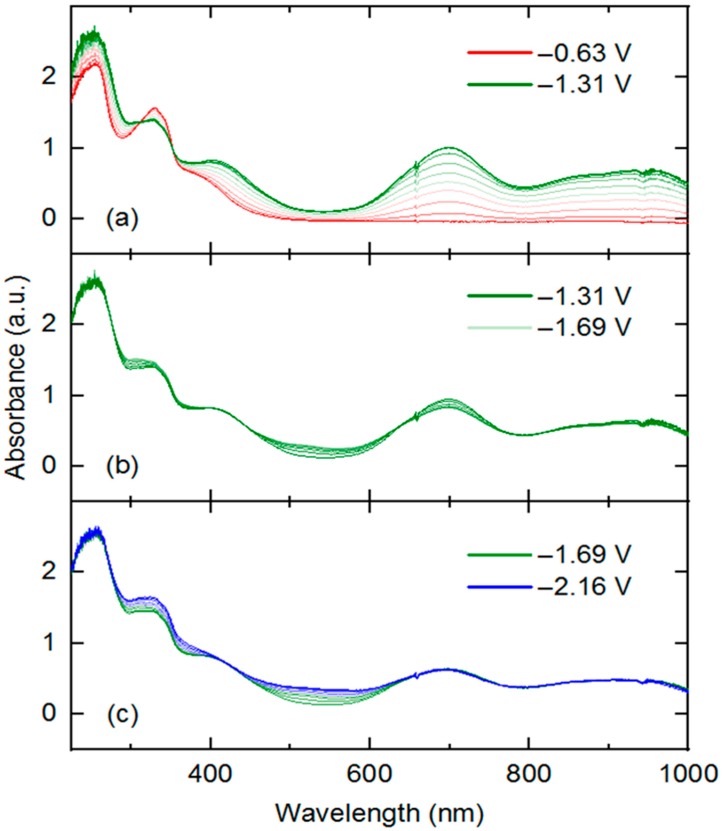
Ultraviolet (UV)-visible-near infrared (NIR) absorption spectra obtained during spectroelectrochemical studies as described in the main text. Initial potentials for each experiment were −0.63 V (panel **a**), −1.31 V (panel **b**), and −1.69 V (panel **c**). Final potentials were −1.31 V (panel **a**), −1.69 V (panel **b**), and −2.16 V (panel **c**). Final potential was held until no spectral changes were reached, indicating full conversion of the thin-layer region to the desired form of the complex.

To gain further insight into the reductions of **3** that are readily accessible via electrochemical methods, we turned to UV-visible spectroelectrochemistry. We took the approach of in situ generation and detection of **4** and other reduced forms of **3** by use of a short-pathlength cuvette cell placed in the beam path of a UV-visible spectrophotometer for real-time data collection during working electrode polarization. With the working electrode polarized at −0.63 V vs. Fc^+/0^, the spectrum of **3** contained in the cell (electrolyte: 0.1 M [NBu_4_][PF_6_] in THF) is virtually identical to that of **3** in pure THF free from supporting electrolyte. However, upon polarization at −1.31 V, the spectrum changes dramatically (Figure 5, panel a), with new features appearing that correspond to those of rhodium(III) bound to reduced dnbpy^∙−^. Notably, isosbestic points were measured at 312 and 352 nm (Figure S22), consistent with clean conversion of **3** to **4** in THF solution under the spectroelectrochemical conditions. However, close comparison of the spectroelectrochemical data (collected in THF electrolyte containing 0.1 M [NBu_4_][PF_6_]) and the earlier UV-visible data collected on **4** (in pure THF) reveals that the λ_max_ values are slightly shifted in the two cases (417 vs. 418, 699 vs. 693, 865 vs. 860, 946 vs. 944 nm, respectively) (see SI, Figure S28). These minor differences are consistent with ligand exchange of chloride in favor of THF, facilitated by 0.1 M [NBu_4_][PF_6_], as similar spectral changes accompany exchange of halide ligands for coordinated solvent (e.g., MeCN) in other rhodium(III) complexes.

To confirm the apparent chemical reversibility observed in cyclic voltammetry for transformation of **3** to **4** (Figure 2) and **4** to **3** (see SI, Figure S16), an experiment was also carried out with an initial potential of −1.31 V and final potential of −0.63 V in an electrolyte solution prepared with **4** (see SI, Figure S26). In this experiment, the evolution of the spectral features detected in the experiment shown in Figure 1a were essentially reversed. This is consistent with clean regeneration of complex **3** from **4** upon electrochemical re-oxidation (isosbestic points at 312 and 352 nm).

Further potential excursions show spectral changes associated with the further reductions of **3**. A potential jump from −1.31 to −1.69 V results in fairly minor changes to the UV-visible spectrum (Figure 5b). Clear isosbestic points were observed at 437, 638, and 800 nm for this reduction event, corresponding to increased spectral absorption toward the blue region (522 nm) and slightly attenuated absorption intensity (peak at 699 nm) toward the longer wavelengths. A further potential excursion to −2.16 V (Figure 5c) results in further increases in absorption toward shorter wavelengths (325 and 522 nm), and virtually no changes in absorption at the longer wavelengths (isosbestic point at 420 nm). Based on the isosbestic behavior, we confirm the interconversion of single species implied by the electrochemical studies carried out in [NBu_4_][PF_6_] [26,40].

However, final potential excursion to −2.56 V results in non-isosbestic spectral evolution (Figure 5c and SI, Figure S25), suggesting formation of multiple speciation products or decomposition of the four-electron reduced species generated from **3**. This behavior is consistent with the irreversible reduction observed at −2.36 V in the voltammetry of **3** and confirms that the quadruply reduced form of **3** is unstable under electrochemical and spectroelectrochemical conditions. Study of the electronic structure of these further reduced intermediates is deserving of future work, especially focusing on the localization of each reduction (metal, ligand, both) and coordination geometry of rhodium (presence or absence of bound chloride or solvent). Computational approaches could be of great use here, as well as further spectroscopic and synthetic/structural investigations.

As reduction of most [Cp*Rh] complexes bearing diimine-type ligands in the presence of acid can give rise to catalytic H_2_ production, we examined electrochemical reduction of **3** in the presence of acid to check for formation of H_2_ (as has been measured for both **1** and **2**). We conducted these studies with **3** in MeCN electrolyte (in order to rely on the well-defined p*K*_a_ scale that is available in this solvent) [18,39]. Addition of 1 atm of H_2_ gas to the headspace of the electrochemical cell results in no major changes to the voltammetric profile, confirming that **3** does not readily serve as a catalyst for H_2_ oxidation (see SI, Figure S17). However, addition of 15 equiv. of buffered Et_3_NH^+^/Et_3_N results in a fully irreversible voltammetric profile, and a modest increase in current density across a broad potential range from −1 V to around −2 V (see SI, Figure S17). Beyond –2 V, there is a significant enhancement in the current flowing in the voltammetry, although at these potentials similar current enhancement is also observed for a rhodium-free electrolyte solution containing only buffered acid.

Bulk electrolysis was then carried out to ascertain the fate of the reducing equivalents transferred to the solution under these acidic conditions. Electrolysis was carried out at −1.75 V, prior to the onset of significant background currents, to reveal the behavior of the reduced metal complexes with acid. In a rhodium-free control experiment, 17.2 C of charge were passed through the electrochemical cell, and a fairly significant amount of H_2_ was generated corresponding to 87% Faradaic efficiency (product H_2_ measured by gas chromatography). The analogous electrolysis carried out with **3** (Figure S18) leads to passage of only 8.2 C of charge, corresponding to 1.95 e^−^ per Rh center. A Faradaic yield of H_2_ of only 5% was measured by gas chromatography, confirming that **3** does not serve as an effective (pre)catalyst for H_2_ evolution under these conditions. 

During electrolysis, the solution of **3** remains homogeneous, but turns a dark red color. To investigate, aliquots of the working solution were removed from the cell following electrolysis, the solvent removed in vacuo, and ^1^H-NMR data collected in CD_3_CN to ascertain the identity of products formed by reaction of reduced **3** with acid. The ^1^H-NMR data reveal that several (≥3) diamagnetic dnbpy containing species are generated, based on the presence of multiple sets of dnbpy-like resonances in the aromatic region (see SI, Figure S19). However, no metal hydride signals were observable in the upfield region near −10 ppm (Figure S20) [25,26], nor were the characteristic signals corresponding to formation of [Cp*H] (e.g., a doublet near 0.5 ppm) detected (Figure S19b,c in SI) [24]. Thus, we conclude that decomposition accompanies reduction and protonation of **3**, resulting in formation of multiple products but no H_2_.

## 3. Discussion

The observation of four one-electron reduction events with complex **3** contrasts with the single two-electron, ECE-type reduction [15] events measured for most other diimine and diphosphine complexes of [Cp*Rh] [8,9,10,24,25,26]. Results from our laboratory suggest that the first reduction is rhodium metal-centered in most of these cases [26,40]. Thus, initial metal-centered reduction generates a transient 19e^−^ rhodium(II) complex that undergoes subsequent ligand dissociation (to a 17e^−^ species) and further reduction. **3** circumvents this more common reactivity by undergoing a first, ligand-centered reduction that leads only to exchange of bound chloride or solvent. As a side note, we have recently chemically prepared an analogous formal rhodium(II) complex bearing a bis(pyridyl) ligand; this complex is a metal-centered radical that circumvents further reduction through use of the bis(pyridyl) ligand that enforces a six-membered metallocyclic ring [40]. Here, we conclude that inclusion of the easily reduced dnbpy ligand and retention of a monodentate ligand in **4** contribute to the ability of the complexes to undergo sequential, one-electron reductions.

Our formulation of **4** as a ligand-centered radical is consistent with prior work from Yellowlees’s group [62] on the nature of reduced species formed by reduction of nitrated bpy complexes of Pt^II^. Specifically, their group found that the LUMO of Pt(dnbpy)Cl_2_ is localized on the dnbpy ligand; on the basis of exhaustive EPR spectroelectrochemistry and computational studies, they further assigned the radical in Pt(dnbpy^∙−^)Cl_2_ to be localized on a single 4-NO_2_-pyridyl ring. The Pt compounds in their study displayed rich hyperfine structure in EPR spectra, enabling this assignment. In our EPR data for **4**, however, no such fine structure is observable. Thus, further work is needed to obtain details regarding the exact localization of unpaired electron density within the conjugated dnbpy system.

Cp*Rh(bpy) undergoes reaction with protons to form [Cp*H] species that are active intermediates in catalytic H_2_ production [23,24]. Therefore, the observation of virtually no H_2_-generating reactivity of reduced forms of **3** with protons is interesting from the perspective of understanding the structure and bonding features that engender catalysis involving H-atom transfer [24,25,26] from [Cp*Rh] complexes. In the chemistry of most [Cp*Rh] complexes, apparently metal-centered reduction is followed by reactivity with protons to form either [Cp*H] complexes [24] or stable rhodium(III) hydride species [25,26]. Based on electrochemical studies of **3** in the presence of Et_3_NH^+^, we conclude that one or more of the reduced forms of **3** undergo reaction with protons, but these do not lead to effective H_2_ generation.

However, involvement in ligand orbitals in reduction events does not necessarily preclude proton reactivity in [Cp*Rh] complexes; rather, our results suggest that the electron-donating and -withdrawing character of ligands on these frameworks must be carefully balanced to accommodate the intermediates that may arise during catalysis. This conclusion is well supported by the prior work implicating a delocalized highest occupied molecular orbital (HOMO) across the metal and ligands in most Cp*Rh(diimine) complexes, and the reactivity of the doubly reduced forms of **1** and **2** with protons towards hydrogen evolution [10].

## 4. Conclusions

We have described the preparation, characterization, and electrochemical properties of [Cp*Rh^III^(dnbpy)Cl]PF_6_ (**3**). This complex displays four one-electron reduction events in organic electrolytes, contrasting with prior work on a similar complex that showed no reductions in aqueous electrolyte. Spectroscopic studies show that the singly reduced complex **4** generated from **3** is best formulated as [Cp*Rh^III^(dnbpy^∙−^)(L)]^+^ where L = chloride or solvent, depending upon the conditions of the experiment. Spectroelectrochemical studies suggest clean interconversion of the various reduced forms, as isosbestic behavior is obtained in the UV-visible spectra associated with controlled potential excursions. However, in contrast to other [Cp*Rh] complexes bearing diimine ligands, electrochemical studies of **3** in the presence of excess Et_3_NH^+^ show that reduction in the presence of this weak acid does not lead to H_2_ production. Taken together, these studies show that [Cp*Rh] complexes, and the reactions that they undergo upon electron transfer, are readily tunable by judicious selection of supporting ancillary ligands. Our ongoing work is examining this strategy to harness the useful properties of this family of compounds.

## 5. Materials and Methods

### 5.1. General Considerations

All manipulations were carried out in dry N_2_-filled gloveboxes (Vacuum Atmospheres Co., Hawthorne, CA, USA) or under N_2_ atmosphere using standard Schlenk techniques unless otherwise noted. All solvents were of commercial grade and dried over activated alumina using a PPT Glass Contour (Nashua, NH, USA) solvent purification system prior to use, and were stored over molecular sieves. All chemicals were from major commercial suppliers and used as received after extensive drying. [Cp*RhCl_2_]_2_ was prepared according to the literature procedure [11]. The 4,4′-dinitro-2,2′-bipyridyl ligand (dnbpy) was prepared with literature methods [42,43,44,45,46,47] from 2,2′-bipyridine (bpy). Deuterated solvents for NMR studies were purchased from Cambridge Isotope Laboratories (Tewksbury, MA, USA); CD_3_CN was dried over molecular sieves. ^1^H-, ^13^C-, ^19^F-, and ^31^P-NMR spectra were collected on 400 or 500 MHz Bruker spectrometers (Bruker, Billerica, MA, USA) and referenced to the residual protio-solvent signal in the case of ^1^H and ^13^C [63]. Heteronuclear NMR spectra were referenced to the appropriate external standard following the recommended scale based on ratios of absolute frequencies (Ξ) [64,65]. ^19^F-NMR spectra are reported relative to CCl_3_F, and ^31^P-NMR spectra are reported relative to H_3_PO_4_. Chemical shifts (δ) are reported in units of ppm and coupling constants (*J*) are reported in Hz. Elemental analyses were performed by Midwest Microlab, Inc. (Indianapolis, IN, USA).

Electronic absorption spectra were collected with an Ocean Optics Flame spectrometer (Ocean Optics, Largo, FL, USA) or a Shimadzu 3600 UV-vis-NIR spectrometer (Shimadzu, Kyoto, Japan), in 1-cm pathlength quartz cuvettes.

Continuous-wave electron paramagnetic resonance were collected at X-band with a Bruker EMX spectrometer using a high-sensitivity perpendicular-mode cavity (4119HS-W1). Temperature control was achieved with an Oxford ESR 900 flow-through cryostat.

### 5.2. X-ray Crystallography

Single-crystal diffraction data were collected with a Bruker APEX-II CCD diffractometer. The Cambridge Crystallographic Data Centre (CCDC) entry 1842459 contains the supplementary crystallographic data for compound **3**. These data can be obtained free of charge via www.ccdc.cam.ac.uk/data_request/cif, or by emailing data_request@ccdc.cam.ac.uk, or by contacting The Cambridge Crystallographic Data Centre, 12, Union Road, Cambridge CB2 1EZ, UK; Fax: +44 1223 336033.

### 5.3. Electrochemistry

Electrochemical experiments were performed in a N_2_-filled glovebox in dry, degassed THF or MeCN. 0.10 M tetra(n-butyl)ammonium hexafluorophosphate ([^n^Bu_4_N]^+^[PF_6_]^−^; Sigma-Aldrich, electrochemical grade) served as the supporting electrolyte. Measurements were carried out with Reference 600+ Potentiostat/Galvanostat (Gamry Instruments, Warminster, PA, USA) using a standard three-electrode configuration. The working electrode was the basal plane of highly oriented pyrolytic graphite (HOPG) (GraphiteStore.com, Buffalo Grove, IL, USA; surface area: 0.09 cm^2^), the counter electrode was a platinum wire (Kurt J. Lesker, Jefferson Hills, PA, USA; 99.99%, 0.5 mm diameter), and a silver wire immersed in electrolyte solution served as a pseudo-reference electrode (CH instruments). The reference was separated from the working solution by a Vycor frit (Bioanalytical Systems, Inc., West Lafayette, IN, USA). Ferrocene (Sigma-Aldrich, St. Louis, MO, USA; twice-sublimed) was added to the electrolyte solution at the end of each experiment; the midpoint potential of the ferrocenium/ferrocene couple (denoted as Fc^+/0^) was used as an external standard for comparison of the recorded potentials.

Concentrations of the analytes for cyclic voltammetry were typically 1 mM. Experiments were typically conducted by first scanning cathodically, then anodically on the return sweep.

Bulk electrolysis experiments were performed in a custom two-chamber electrochemical cell equipped with connections to achieve gas-tight operation. The working electrode was a HOPG plate (Graphitestore.com, Buffalo Grove, IL, USA; surface area: 10 cm^2^). 10 equiv. of ferrocene served as the sacrificial reductant.

### 5.4. Spectroelectrochemistry

Spectroelectrochemisty was carried out in the same glovebox as described above (N_2_ atmosphere), with 0.10 M [^n^Bu_4_N][PF_6_] in THF as electrolyte. A thin layer quartz cell was used with a Teflon cap for housing the electrodes (ALS Co., Ltd., Tokyo, Japan; path length: 1.0 mm). The working electrode was a platinum mesh/flag electrode covered with a PTFE shrink tube up to the flag, and the counter electrode was a platinum wire (ALS Co., Ltd.).

### 5.5. Gas Chromatography

Gas chromatography were collected with a Shimadzu GC-2014 CustomGC. The instrument was calibrated with a standard checkout gas mixture (Agilent 5190-0519, Santa Clara, CA, USA) prior to experimental runs to obtain quantitative data for H_2_ and other gases. Calibration curves over a range of 100–10,000 ppm were constructed with prepared mixture of H_2_ and N_2_ to enable H_2_ quantification.

### 5.6. Preparation of [Cp*Rh(4,4′-dinitro-2,2′-bipyridyl)Cl]PF_6_ *(**3**)*

THF solutions of dnbpy (0.0249 g, 0.101 mmol, 2 equiv.) and AgPF_6_ (0.026 g, 0.103 mmol, 2 equiv.) were added in sequence to a suspension of [Cp*RhCl_2_]_2_ (0.0314 g, 0.051 mmol, 1 equiv.) in THF. A gradual color change occurred over 20 min from dark red to bright yellow and a precipitate appeared. This suspension was filtered over Celite, and a homogeneous yellow solution was obtained. Trituration with approximately 50 mL Et_2_O resulted in formation of a bright yellow solid. The solution was decanted and excess solvent pumped off to obtain **3** (0.0294 g, 44% yield). Crystals suitable for X-ray diffraction analysis were obtained by vapor diffusion of Et_2_O into a solution of **3** in acetonitrile. ^1^H-NMR (500 MHz, CD_3_CN) δ 9.31 (d, ^4^*J*_H,H_ = 2.3 Hz, 2H), 9.23 (d, ^3^*J*_H,H_ = 6.0, 2H), 8.52 (dd, ^4^*J*_H,H_ = 2.2, ^3^*J*_H,H_ = 6.0 Hz, 2H), 1.73 (s, 15H) ppm. ^13^C{^1^H}-NMR (126 MHz, CD_3_CN) δ 156.90, 156.49, 155.49, 123.01, 119.36, 99.69 (d, ^1^*J*_C,Rh_ = 8.26 Hz), 9.26 ppm. ^31^P{^1^H}-NMR (162 MHz, CD_3_CN) δ −146.88 (sept, ^1^*J*_P,F_ = 706.3 Hz). ^19^F-NMR (376 MHz, CD_3_CN) δ −72.9 (d, ^1^*J*_F,P_ = 707.0 Hz). Electronic absorption spectrum (THF): 239 (36,400), 323 (13,000), 365 nm (5300 M^−1^ cm^−1^). Electrospray ionization mass spectrometry (ESI-MS) (positive) *m*/*z*: 519.03 [**3** − PF_6_^−^]^+^.

Elemental analysis for a sample of **3** found 37.20% carbon, 4.43% hydrogen, and 7.78% nitrogen. Calculated values were 36.14%, 3.18%, and 8.43% respectively. Inclusion of trace THF associated with the isolated powder (0.3 eq.) provides the appropriate analysis values of 37.20%, 3.46%, and 8.13% respectively. These are within 0.4% error of the analytical results and are consistent with isolation of solid **3** from THF.

### 5.7. Generation and Isolation of [Cp*Rh(4,4′-dinitro-2,2′-bipyridyl)(L)]^+^ *(**4**)*

A solution of cobaltocene (0.0142, 0.075 mmol, 2 equiv.) in THF was added dropwise to a THF solution of **3** (0.0250 g, 0.037 mmol, 1 equiv.) while stirring. The color immediately changed from a light yellow to a dark green. After stirring for 10 m, the solution was pumped down to obtain a dark solid. This was washed with pentane and Et_2_O. The resulting solid was again dissolved in THF and filtered over Celite to obtain a dark green homogeneous solution. Removal of volatiles gave **4** as a dark green solid (0.0156 g, 62% yield). Electronic absorption spectrum (THF): 264 (28,500), 304 (6000), 418 (5200), 693 (13,000), 860 (6000), 944 (7800 M^−1^ cm^−1^).

## Figures and Tables

**Table 1:** Cyclic voltammetry data for **3**. Conditions: [**3**] = 10^−3^ M; scan rate: 100 mV/s; electrolyte: 0.1 M [NBu_4_][PF_6_] in THF.

Redox Event	E_1/2_ (V)	ΔE_p_ (V)	E_p,c_ (V)	E_p,a_ (V)
**A**	−0.94	0.20	−1.04	−0.84
**B**	−1.44	0.18	−1.53	−1.35
**C**	−1.89	0.17	−1.98	−1.81
**D**	-	-	−2.36	-

## Supplementary Materials

The following are available online: NMR spectra; crystallographic details; electronic absorption spectra; electrochemical, spectroelectrochemical, and gas chromatography data (PDF); cartesian coordinates (XYZ).
