# Supplementary material for: Single-Electron Redox Chemistry on the [Cp*Rh] Platform Enabled by a Nitrated Bipyridyl Ligand
Source: Molecules. 2018 Nov 2;23(11):2857. doi: 10.3390/molecules23112857 (PMC6278249; doi:10.3390/molecules23112857)
Supplement: Supplementary file 1 [file molecules-23-02857-s001.zip › SI_For_Publication/SI.pdf]

Supporting Information for:

Single-Electron Redox Chemistry on the [Cp*Rh] Platform Enabled by a Nitrated Bipyridyl Ligand

William N.G. Moore, Wade C. Henke, Davide Lionetti, Victor W. Day, and James D. Blakemore,*

Department of Chemistry, University of Kansas, 1251 Wescoe Hall Drive, Lawrence, Kansas 66045, United States

*E–mail: blakemore@ku.edu (J.D.B.)

Contents

**NMR Spectroscopy**	S3
**Figure S1.** ^{1}H NMR spectrum of **3**	S3
**Figure S2.** $^{13}C\{^{1}H\}$NMR spectrum of **3**	S3
**Figure S3.** $^{31}P\{^{1}H\}$NMR spectrum of **3**	S3
**Figure S4.** ^{19}F NMR spectrum of **3**	S4
**Figure S5.** ^{1}H NMR spectrum of isolated **4**	S4
**Mass Spectrometry**	S5
**Figure S6.** Mass spectrum of **3**	S5
**Optical Spectroscopy**	S6
**Figure S7.** UV–vis spectrum of **3**	S6
**Figure S8.** UV–vis and near–IR spectra of isolated **4**	S7
**Electrochemistry**	S8
**Figure S9.** Cyclic voltammetry (CV) of **3**	S8
**Figure S10.** Solvent–dependent CV of **3**	S8
**Table S1.** Tabulated CV of data for **3**	S9
**Table S2.** Peak positions for **3**	S9
**Figure S11.** CV of **3** with 0.1 M [$^{n}Bu_4$][Cl] in THF	S10
**Figure S12.** Gaussian fit of DPV of **3** in THF	S11
**Table S3.** Tabulated Gaussian fit data	S11
**Figure S13.** Scan rate dependence of **3** (couple A)	S12
**Figure S14.** Scan rate dependence of **3** (couple B)	S12
**Figure S15.** Scan rate dependence of **3** (couple C)	S13
**Figure S15.** CV overlay of **3** and **4**	S13
**Table S4.** Tabulated CV data for **3** and **4**	S14
**Table S3.** Et_3NH^+/Et_3N buffer addition to **3** in CV	S14

Bulk Electrolysis S15
Table S18. Chronoamperometry experiments for bulk electrolysis S15
Figure S19a. ^{1}H NMR overlay of bulk solution with **3** and free ligand S16
Figure S19b. ^{1}H NMR of bulk solution (aromatic region) S16
Figure S19c. ^{1}H NMR of bulk solution (aliphatic region) S16
Figure S20. ^{1}H NMR of bulk solution extended range S17

Spectroelectrochemistry S18
Figure S21. Representative chronoamperometry S18
Figure S22. UV–vis spectra during polarization after couple A S18
Figure S23. UV–vis spectra during polarization after couple B S19
Figure S24. UV–vis spectra during polarization after couple C S19
Figure S25. UV–vis spectra during polarization after couple D S20
Figure S26. UV–vis spectra during polarization back positive of couple A S20
Figure S27. UV–vis spectral overlay of 5 accessible forms S21
Figure S28. UV–vis spectral overlay of **4** with electrochemically reduced **3** S21

Crystallographic Information S22
Refinement Details for **3** S22
Table S29: Crystal and Refinement Data S23
Special Refinement Details for 3. S24
Figure S30. Full solid-state structure of **3** S24
Figure S31. Isolated solid-state structure of **3** S25

References S26

NMR Spectroscopy

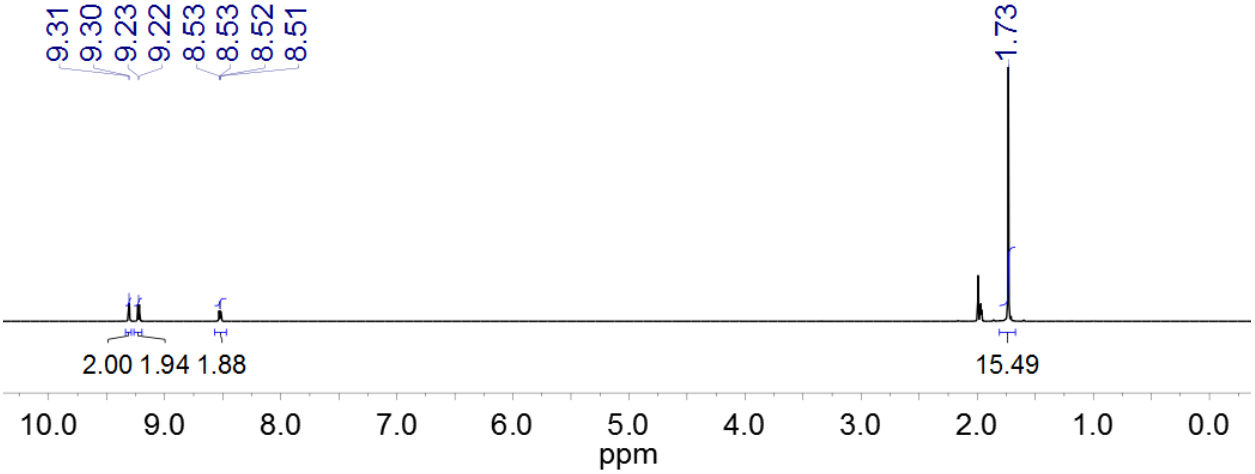

Figure S1. ^{1}H NMR spectrum (500 MHz, CD_3CN) of **3**.

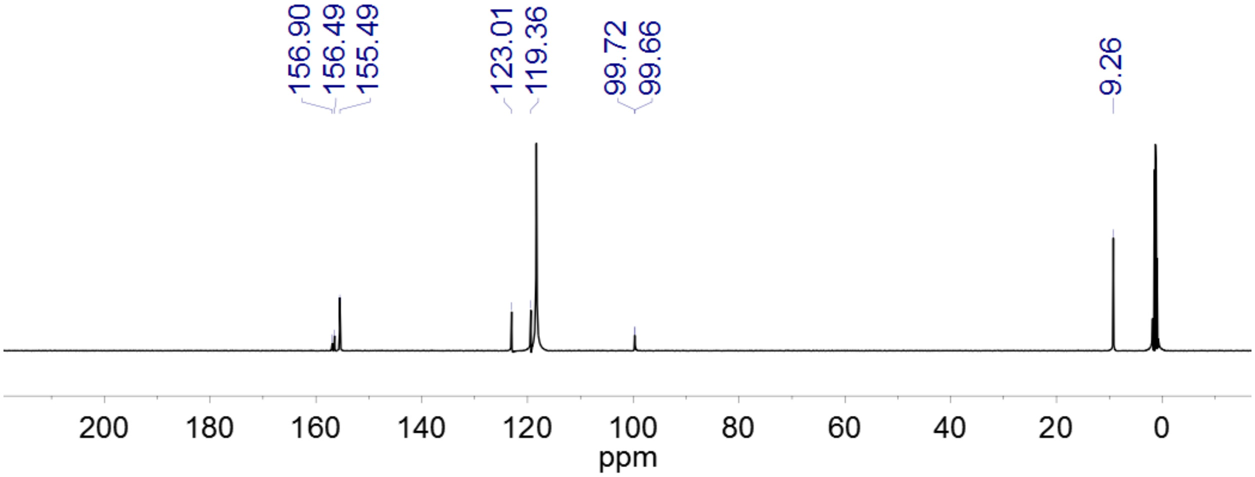

Figure S2. $^{13}C\{^{1}H\}$ NMR spectrum (126 MHz, CD_3CN) of **3**.

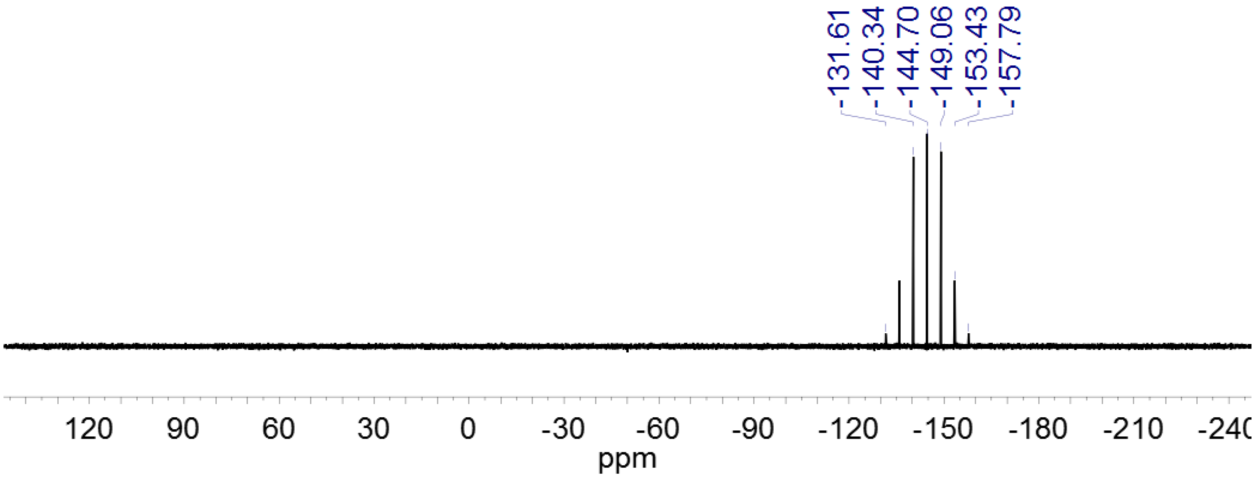

Figure S3. $^{31}P\{^{1}H\}$ NMR spectrum (162 MHz, CD_3CN) of **3**.

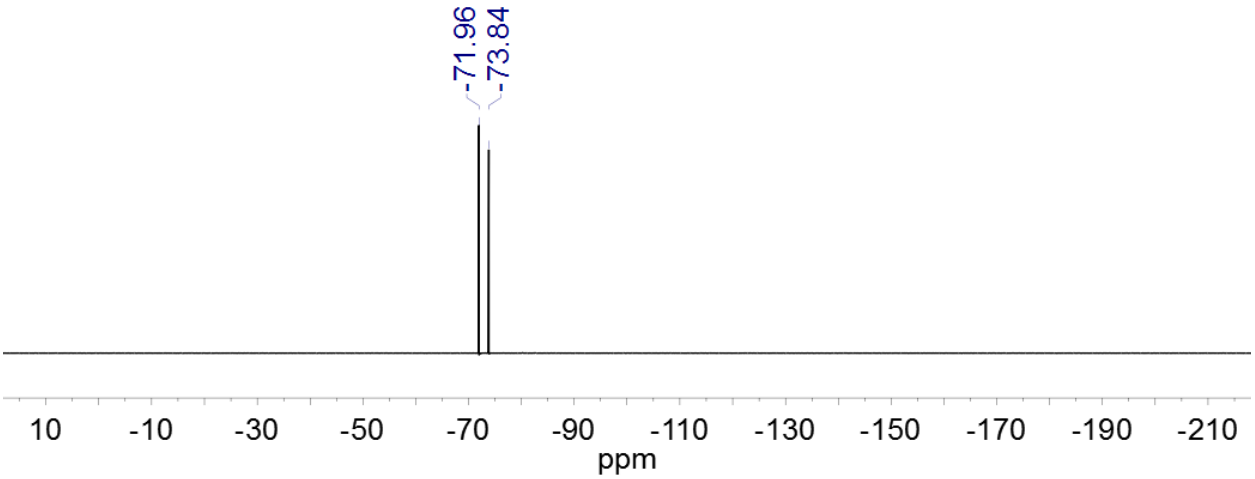

Figure S4. ^{19}F NMR spectrum (376 MHz, CD_3CN) of **3**.

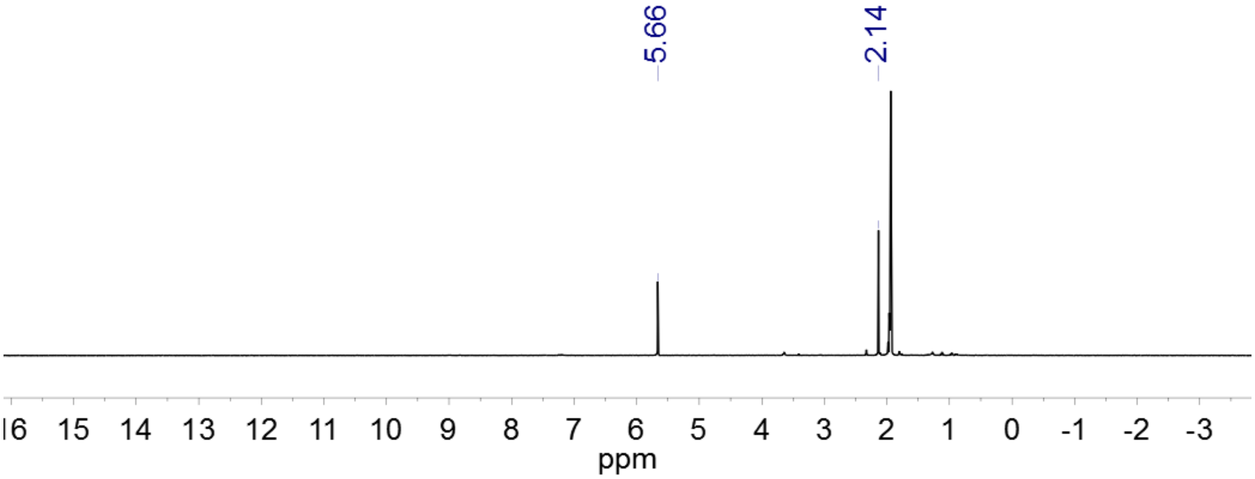

Figure S5. ^{1}H NMR spectrum (400 MHz, CD_3CN) of aliquot from reduction of **3**. Minor trace impurities: water, THF, Et_2O, toluene, acetonitrile, and pump oil. Cobaltocenium visible at δ = 5.66 ppm.

Mass Spectrometry

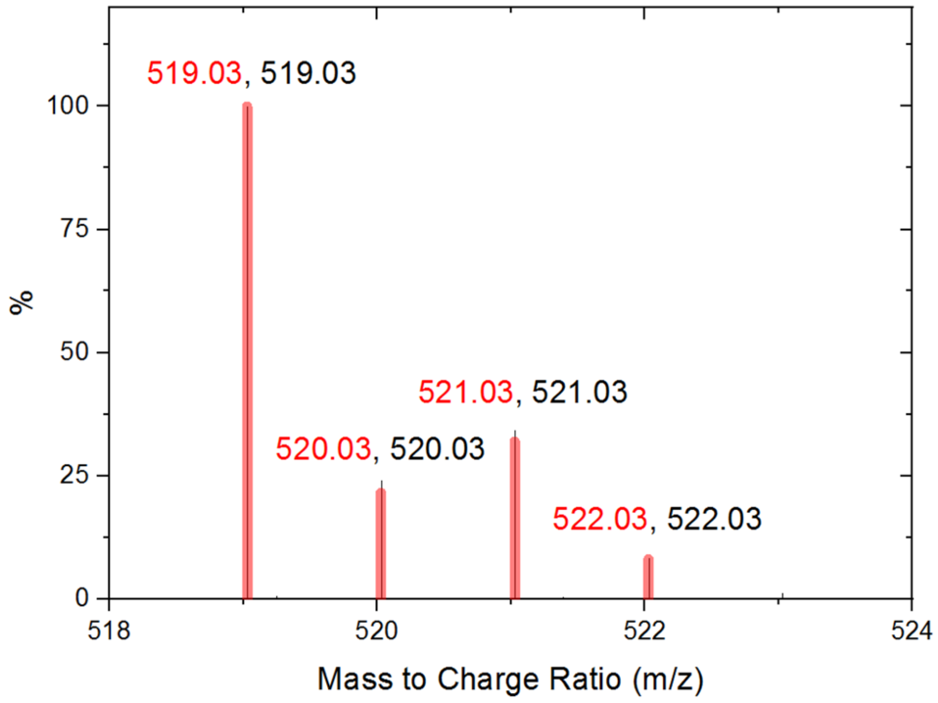

Figure S6. Mass spectrometry of **3**. Comparison overlay of predicted (red) and experimental (black) mass spectra in MeCN.

Optical Spectroscopy

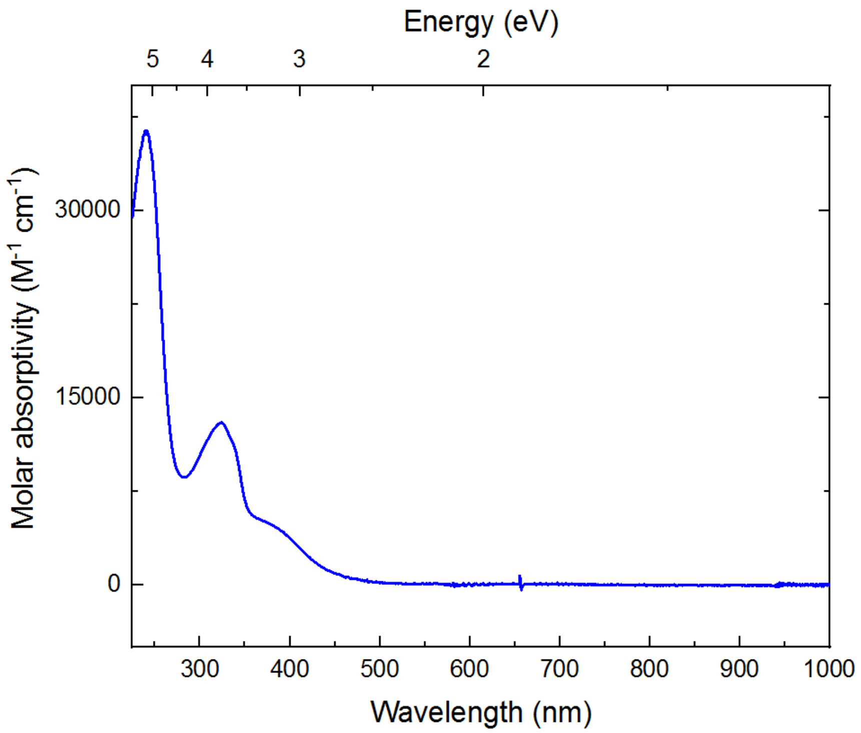

Figure S7. Electronic absorption spectrum of **3** in THF: 263 (36400), 323 (13000), 365 (5300 M^{-1} cm^{-1}).

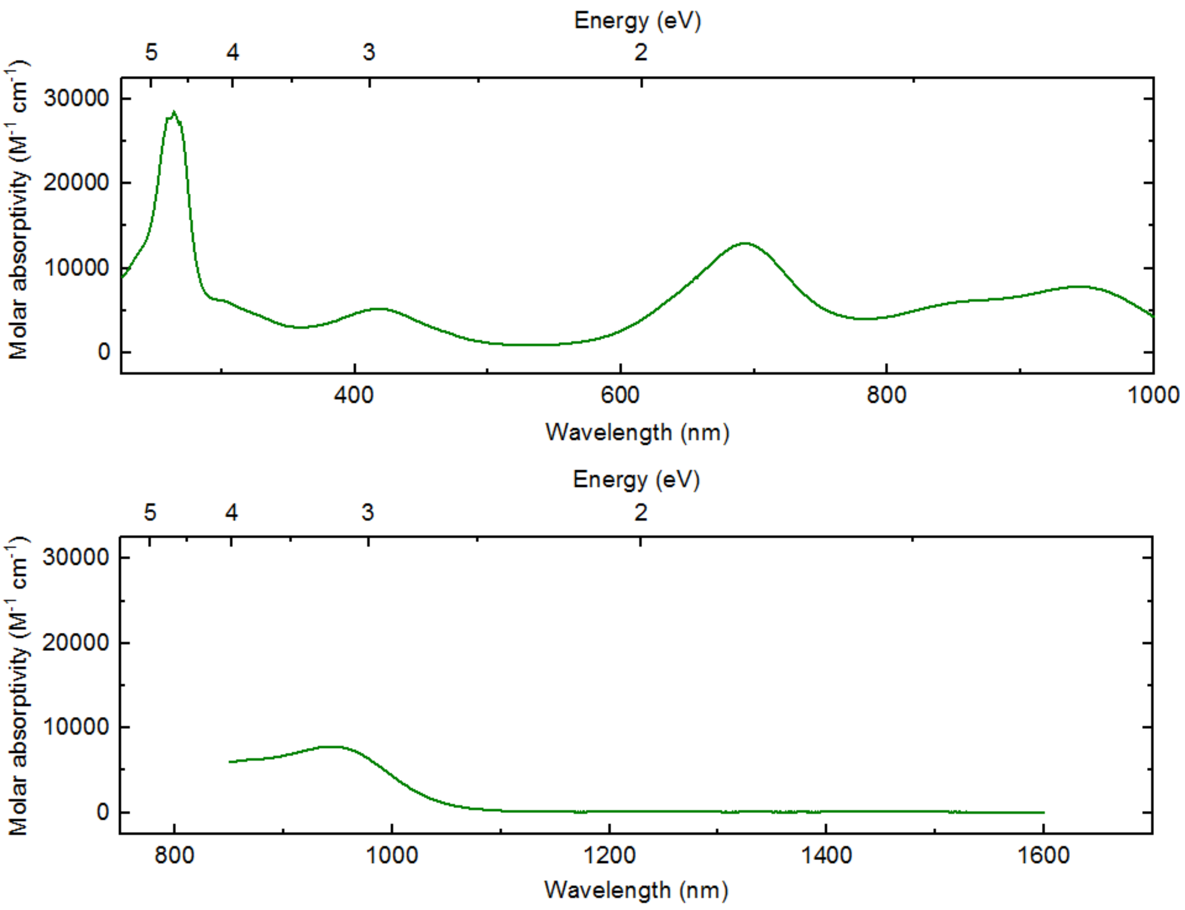

Figure S8. Electronic absorption of isolated **4** in the UV-visible region (top panel) and near infrared (NIR) region (bottom panel): 265 (28500), 300 (6100), 420 (5200), 694 (13000), 860 (6100), and 945 (7800). Spectra were taken with the same 0.1 mM solution in a Schlenk cuvette under an atmosphere of N_2. The near IR spectrum was collected with a Shimadzu 3600 UV-vis-NIR Spectrophotometer.

Electrochemistry

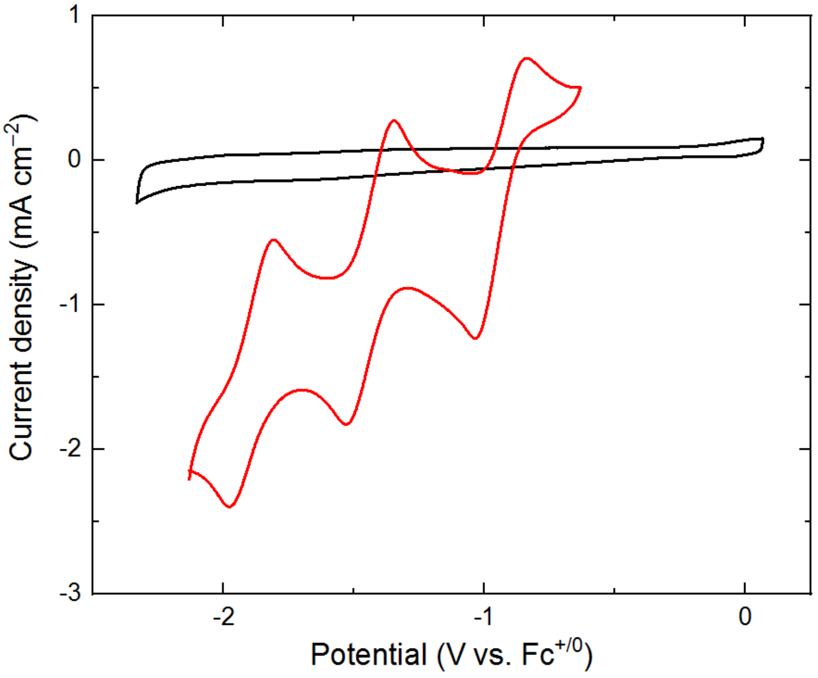

Figure S9. Cyclic voltammetry of **3** (THF, 0.1 M [^{n}Bu$_4$N][PF$_6$], 100 mV/s; red line) overlayed on background response (black line). Second complete cycles shown for both voltammograms.

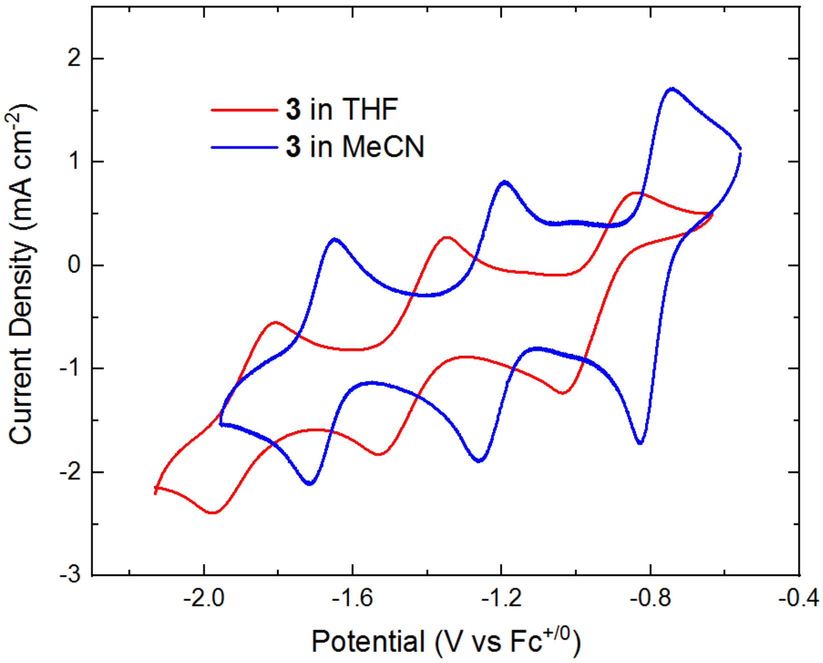

Figure S10. Cyclic voltammetry of **3** in THF and MeCN with 0.1 M [^{n}Bu$_4$N][PF$_6$] as supporting electrolyte at a 100 mV/s scan rate.

	THF		Acetonitrile	
Couple	$E_{1/2}$	ΔE_p	$E_{1/2}$	ΔE_p
A	−0.94	0.20	−0.79	0.09
B	−1.44	0.18	−1.23	0.07
C	−1.89	0.17	−1.68	0.07
D	−2.19	0.35	−2.07	0.12

Table S1. Tabulated cyclic voltammetric data (midpoint potentials, $E_{1/2}$, and peak–to–peak separations, ΔE_p) for **3** in THF and MeCN with 0.1 M [$^nBu_4N$][PF_6] as supporting electrolyte at a 100 mV/s scan rate.

Solvent	$E_{pc}^{(A)}$	$E_{pa}^{(A)}$	$E_{pc}^{(B)}$	$E_{pa}^{(B)}$	$E_{pc}^{(C)}$	$E_{pa}^{(C)}$	$E_{pc}^{(D)}$	$E_{pa}^{(D)}$
THF	–1.04	–0.84	–1.53	–1.35	–1.98	–1.81	–2.36	~ –2.0
Acetonitrile	–0.83	–0.74	–1.26	–1.19	–1.72	–1.65	–2.12	~ –2.0

Table S2. Cyclic voltammetric peak positions (cathodic, $E_{p,c}$, and anodic, $E_{p,a}$) for **3** in THF and MeCN with 0.1 M [$^nBu_4N$][PF_6] as supporting electrolyte at a 100 mV/s scan rate.

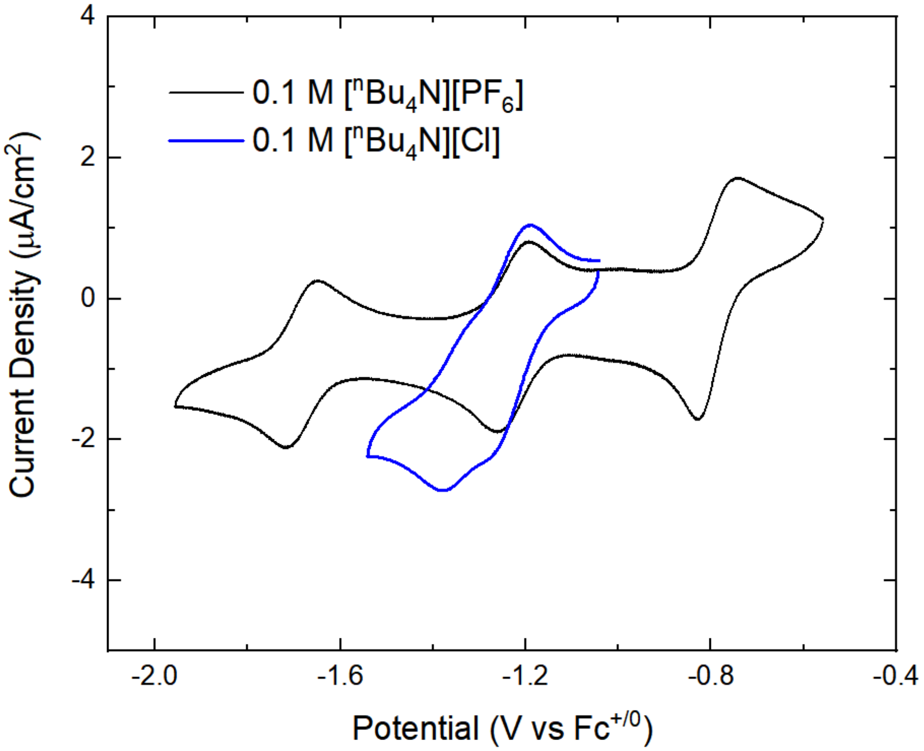

Figure S11. Cyclic voltammetry of **3** in acetonitrile with 0.1 M [$^{n}Bu_4N$][PF_6] and [$^{n}Bu_4N$][Cl] respectively. Reduction events occur at –1.28 and –1.38 V vs $Fc^{+/0}$. Single oxidation occurs at –1.19 V.

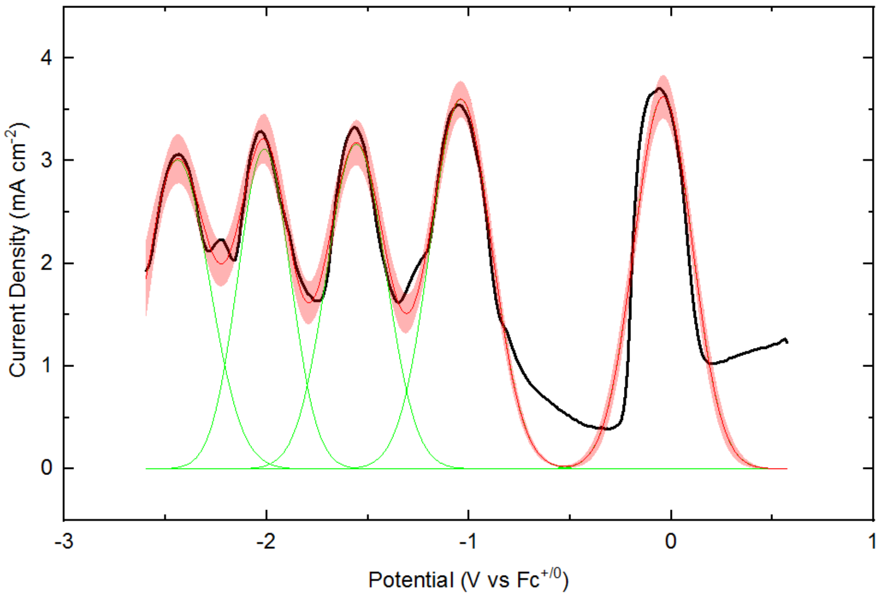

Figure S12. Differential pulse voltammetry (THF, 0.1 M [$^{n}Bu_4N$][PF_6], 10 mV step size) of **3** with one equivalent of ferrocene (black line). Gaussian fit of the five peaks shown in green and red lines. Associated uncertainty at 95% included in light red.

Couple	Peak area ratio w.r.t. $Fc^{+/0}$ peak	Fit peak area
D	0.9 ± 0.1	1.02 ± 0.10
C	0.8 ± 0.1	1.00 ± 0.10
B	0.9 ± 0.1	1.16 ± 0.06
A	1.1 ± 0.1	1.37 ± 0.03
$Fc^{+/0}$	1.0 ± 0.1	1.30 ± 0.04

Table S3. Tabulated differential pulse voltammetry data for **3** in THF electrolyte at scan rate of 100 mV/s.

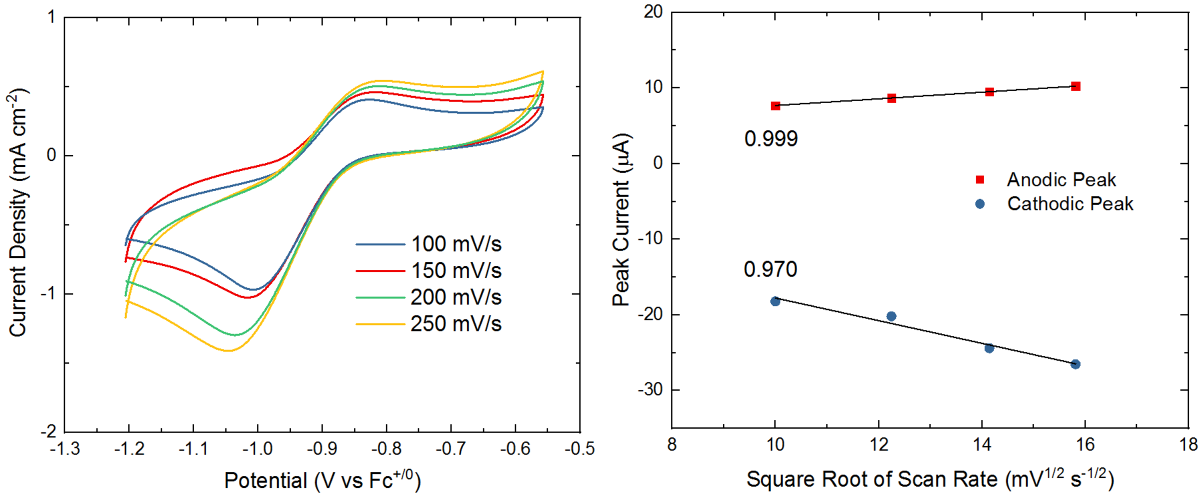

Figure S13. Scan rate dependence of couple A indicating free diffusion.

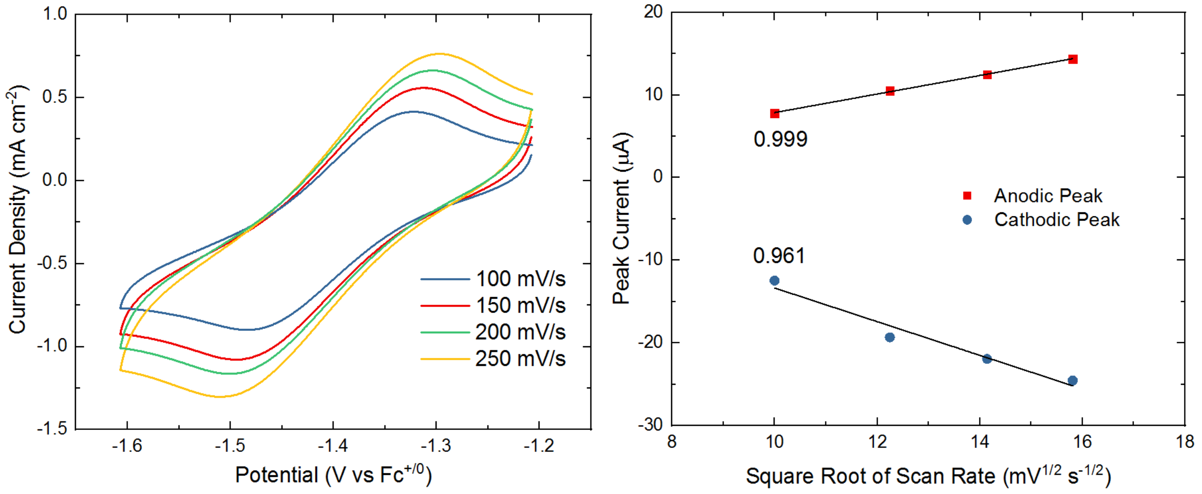

Figure S14. Scan rate dependence of couple B indicating free diffusion.

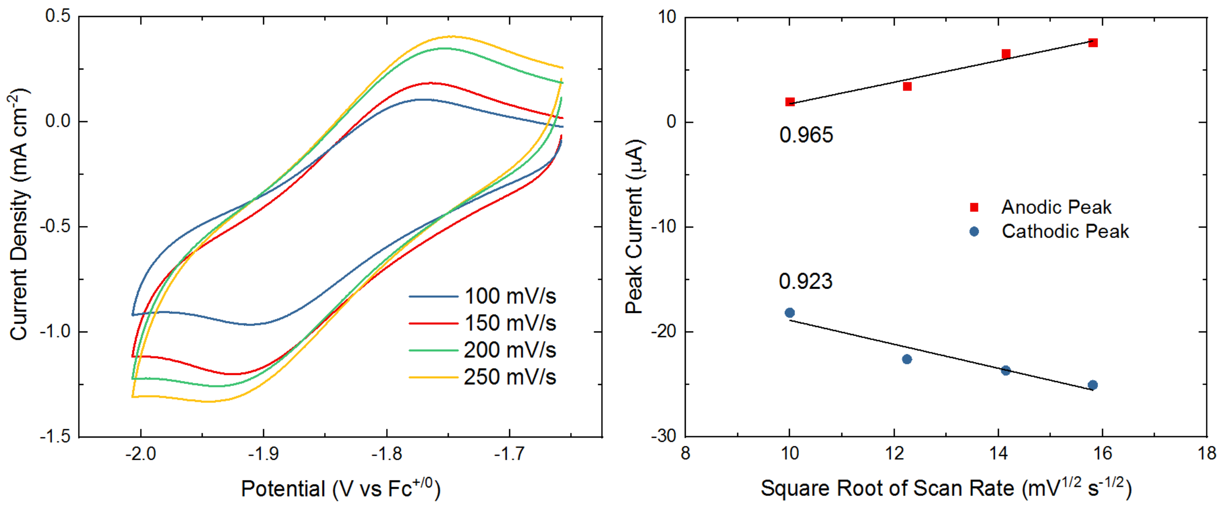

Figure S15. Scan rate dependence of couple C indicating semi–free diffusion. Small discrepencies indicate marginal instability of triply reduced **3**.

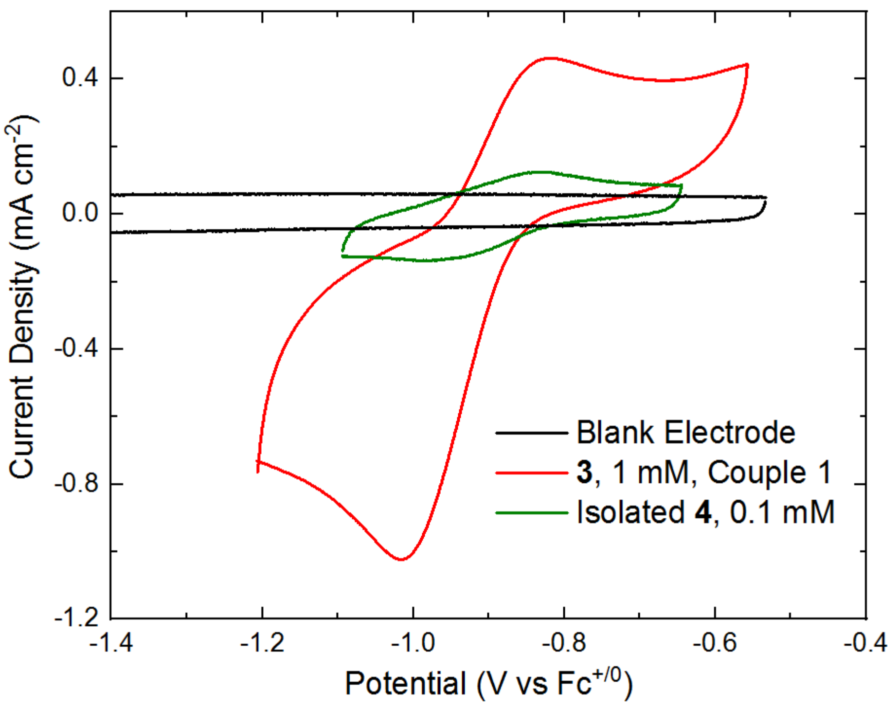

Figure S16. Cyclic voltammetry (THF, 0.1 M [$^{n}Bu_4N$][PF_6]) of reduction product **4** in comparison with the starting **3**.

	$E_{p,c}$(A)	$E_{p,a}$(A)	$E_{1/2}$	ΔE_p
**3**	–1.04	–0.84	–0.94	0.20
Isolated **4**	–1.02	–0.84	–0.93	0.18

Table S4. Tabulated cyclic voltammetry data for **3** and **4** in THF electrolyte at scan rate of 100 mV/s.

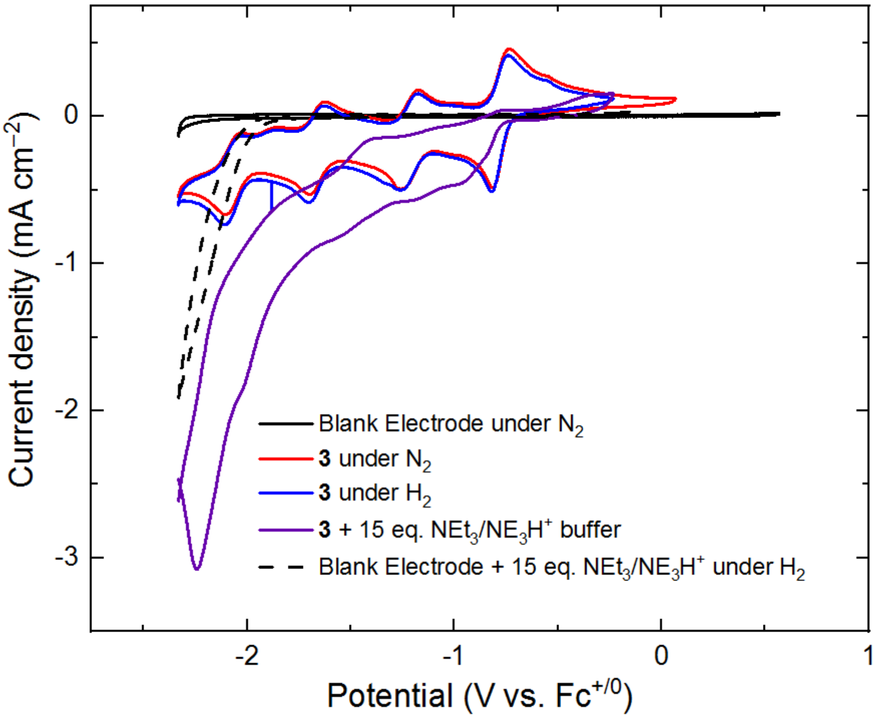

Figure S17. Cyclic voltammetry studies in MeCN with addition of triethylamine (NEt_3) and triethylammonium bromide ($[Et_3NH][Br]$) buffer under an atmosphere of H_2.

Bulk Electrolysis

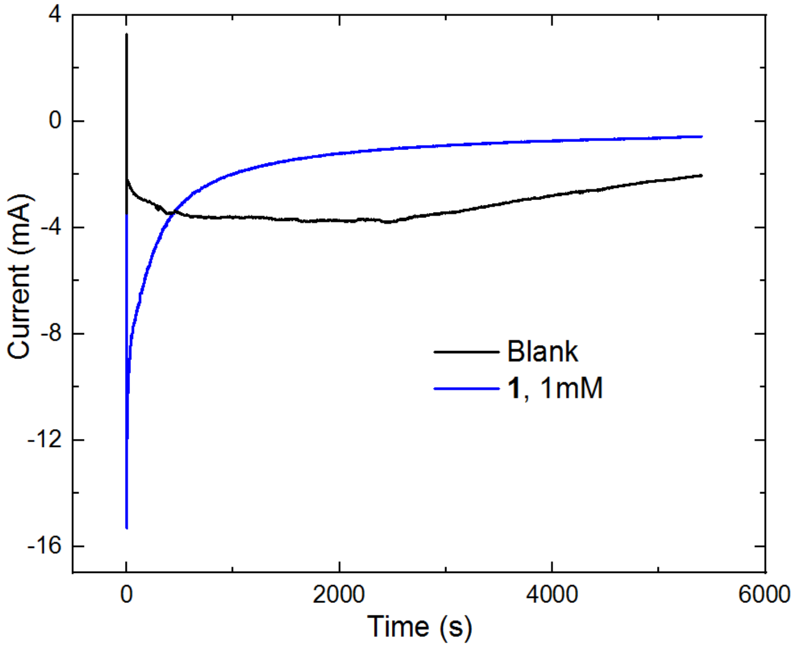

Figure S18. Chronoamperometry experiments conducted during bulk electrolyses. Polarization at −1.75 V vs $Fc^{+/0}$. Ten equivalents of ferrocene included as sacrificial reductant, and 10 equivalents of $[Et_3NH][Br]$ added as the acid. Supporting electrolyte was 0.1 M $[^{n}Bu_4N][PF_6]$ in each case.

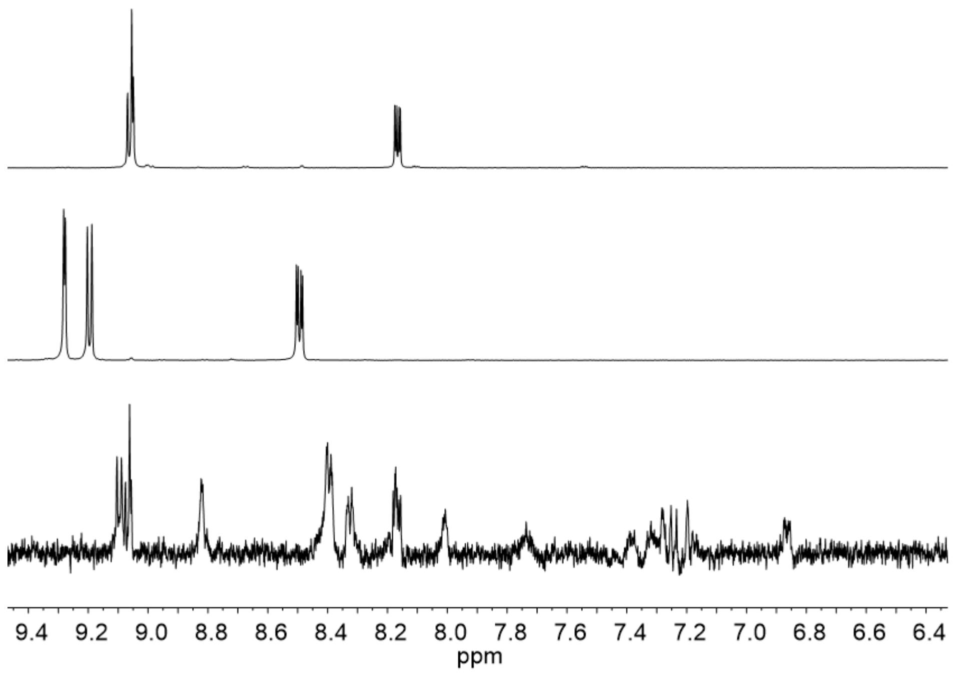

Figure S19a. ^{1}H NMR spectra (400 MHz, CD_3CN) of free ligand (top panel), pure **3** (middle panel), and pumped down bulk electrolysis solution (bottom panel). Alophatic region cut out for clarity due to large [$^{n}Bu_4N$][PF_6] peaks.

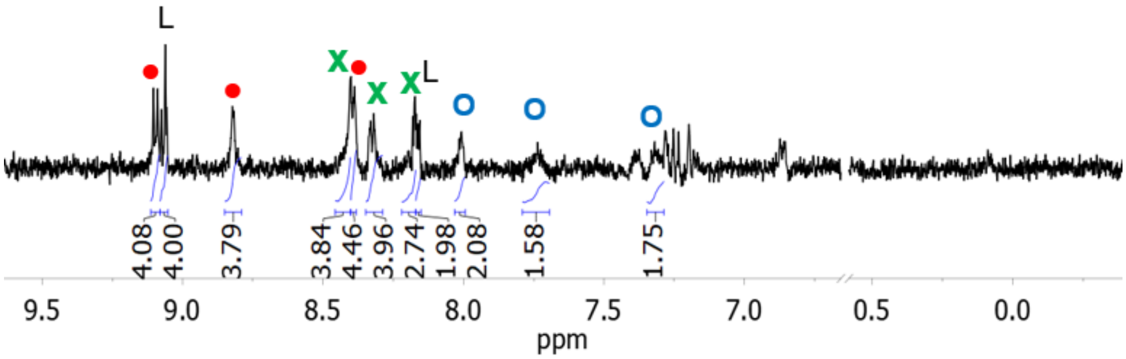

Figure S19b. ^{1}H NMR spectra (400 MHz, CD_3CN) of pumped down bulk electrolysis solution showing three sets of dnbpy peaks (●, **X**, **O**) integrating approximately 1:1:1 in addition to two signals corresponding to free ligand (**L**, 2:1 due to overlap).

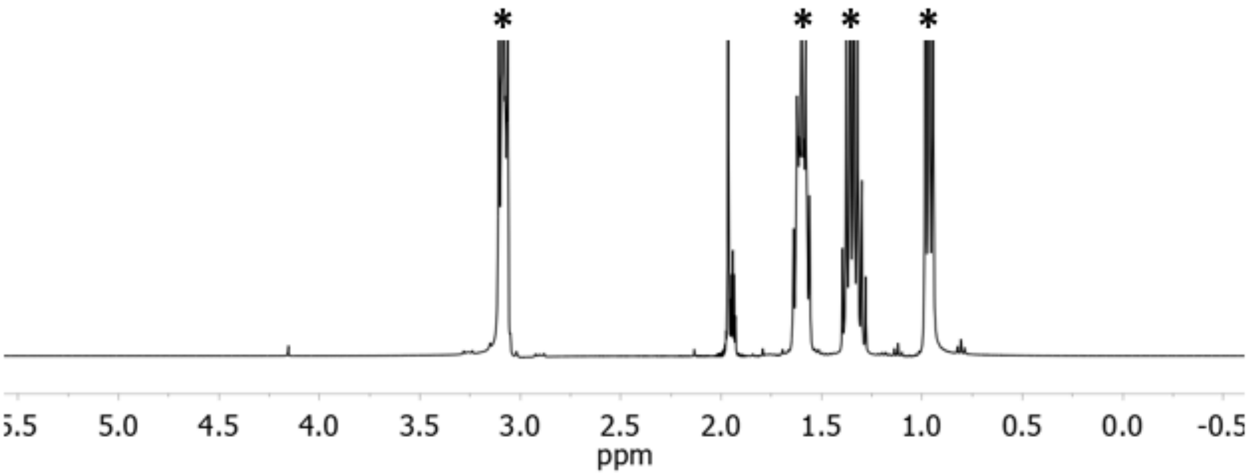

Figure S19c. ^{1}H NMR (CD_3CN, 400 MHz) showing the aliphatic region of sample obtained from bulk electrolysis solution following removal of solvent *in vacuo*. Four sharp peaks visible (✱) corresponding to tetrabutylammonium and triethylammonium.

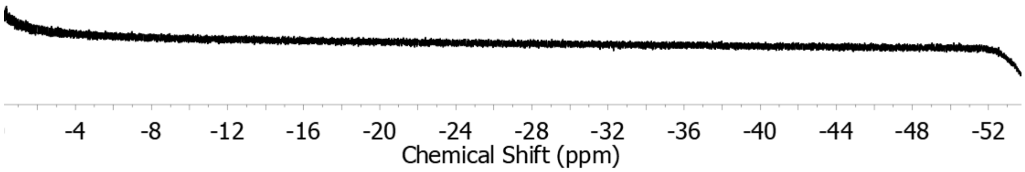

Figure S20. ^{1}H NMR spectrum (400 MHz, CD_3CN) of aliquot from bulk electrolysis cell, pumped down. No hydride signals visible.

Spectroelectrochemistry (SEC)

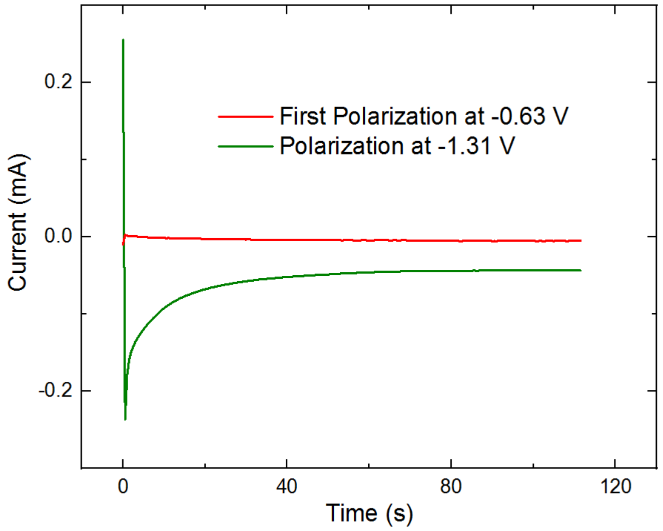

Figure S21. Chronoamperometry experiments for polarization positive and negative of the first redox couple.

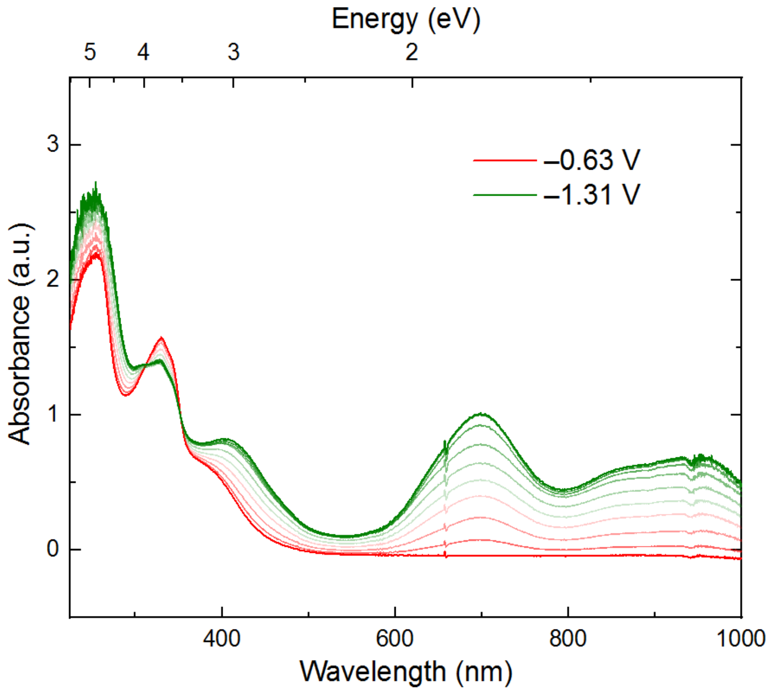

Figure S22. Electronic absorption spectra of **3** selected at regular intervals during polarization (initial: −0.63 V, final: −1.31 V vs. to $Fc^{+/0}$). Isosbestic points are located at 312 and 352 nm.

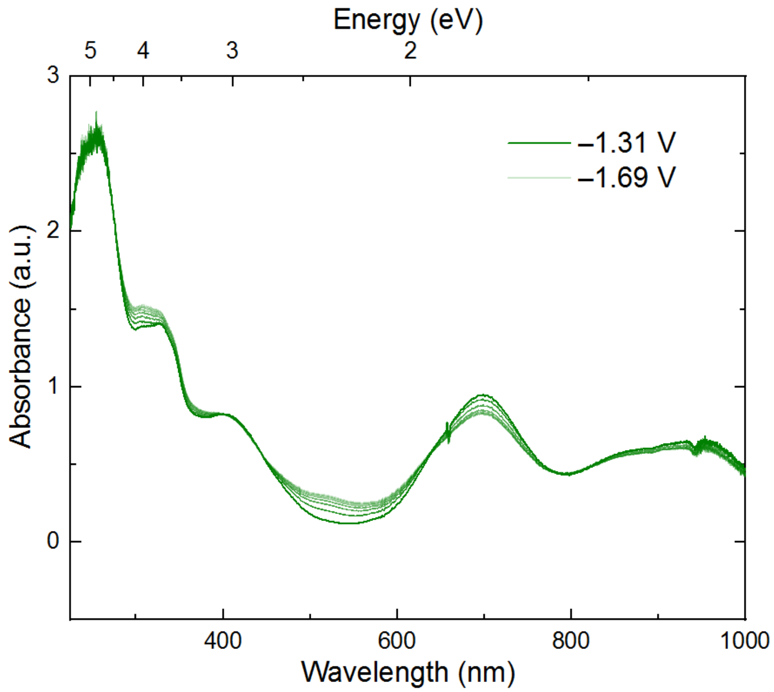

Figure S23. Electronic absorption spectra selected at regular intervals during polarization (initial: −1.31 V, final: −1.69 V vs. $Fc^{+/0}$). Isosbestic points are located at 437, 638, and 800 nm.

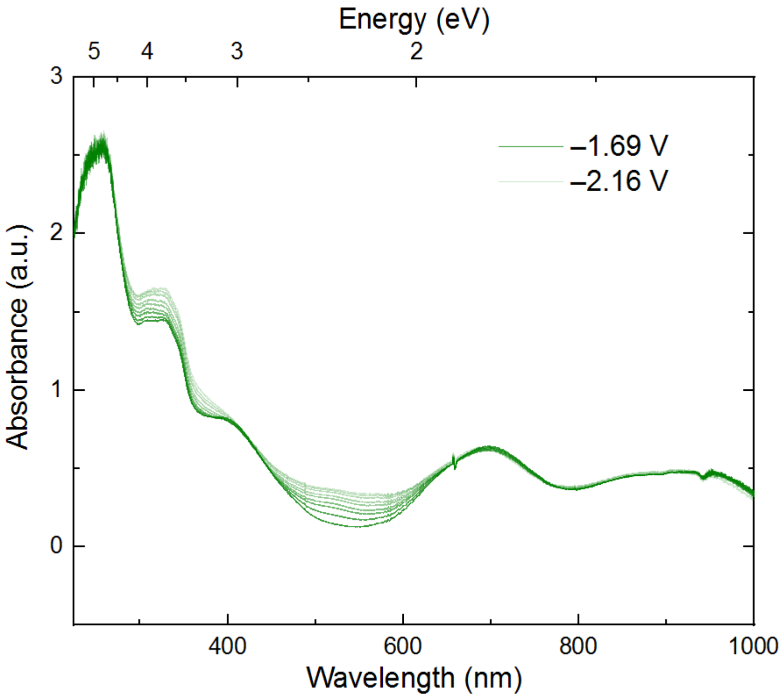

Figure S24. Electronic absorption spectra selected at regular intervals during polarization (initial: −1.69 V, final: −2.16 V vs. $Fc^{+/0}$). Isosbestic point located at 420 nm.

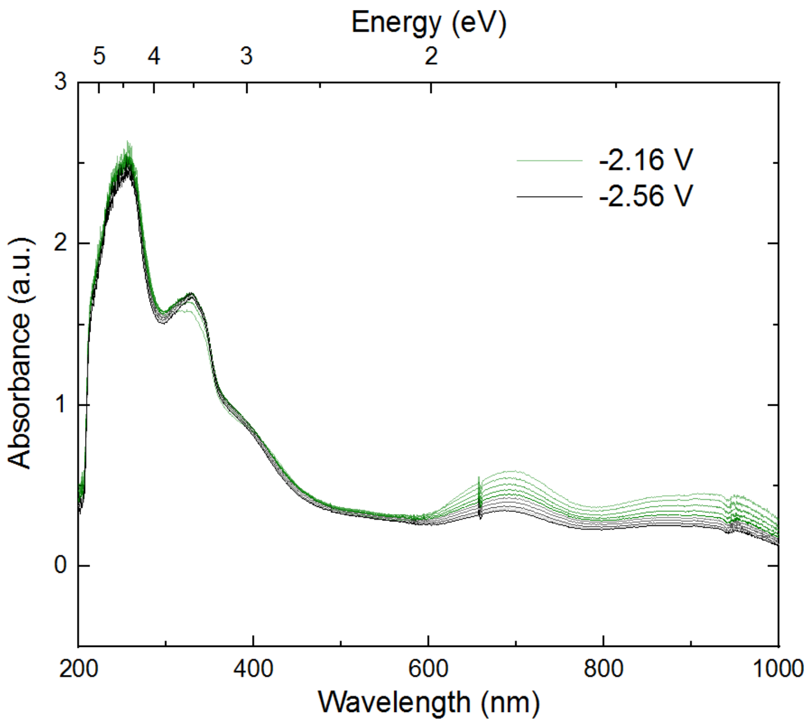

Figure S25. Electronic absorption spectra selected at regular intervals during polarization (initial: −2.16 V, final: −2.56 V vs. $Fc^{+/0}$).

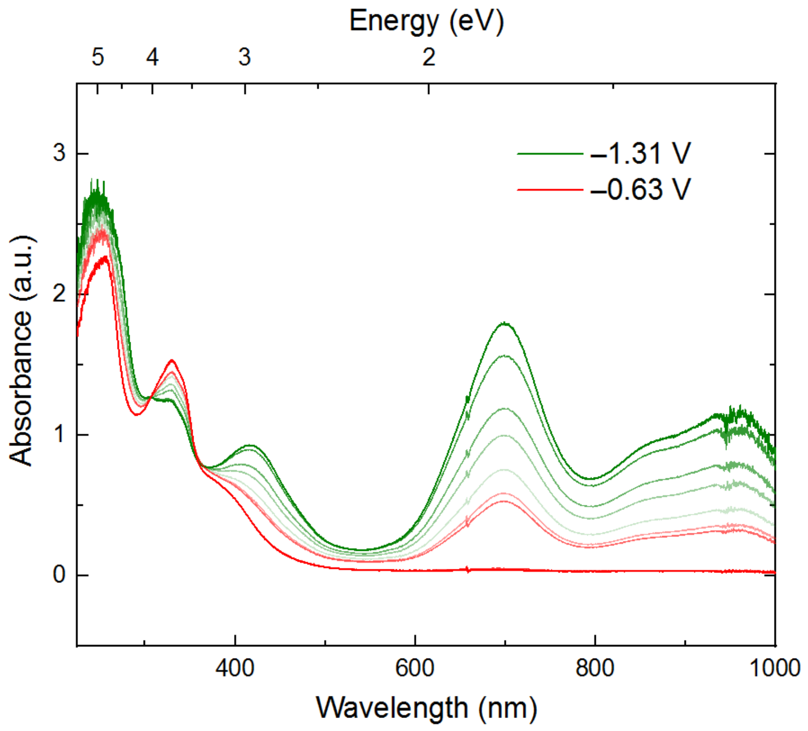

Figure S26. Electronic absorption spectra selected at regular intervals during polarization (initial: −1.31 V, final: −0.63 V vs. $Fc^{+/0}$). Distance from working to counter electrode altered from 10 to 1 mm. Isosbestic points remain at 312 and 352 nm.

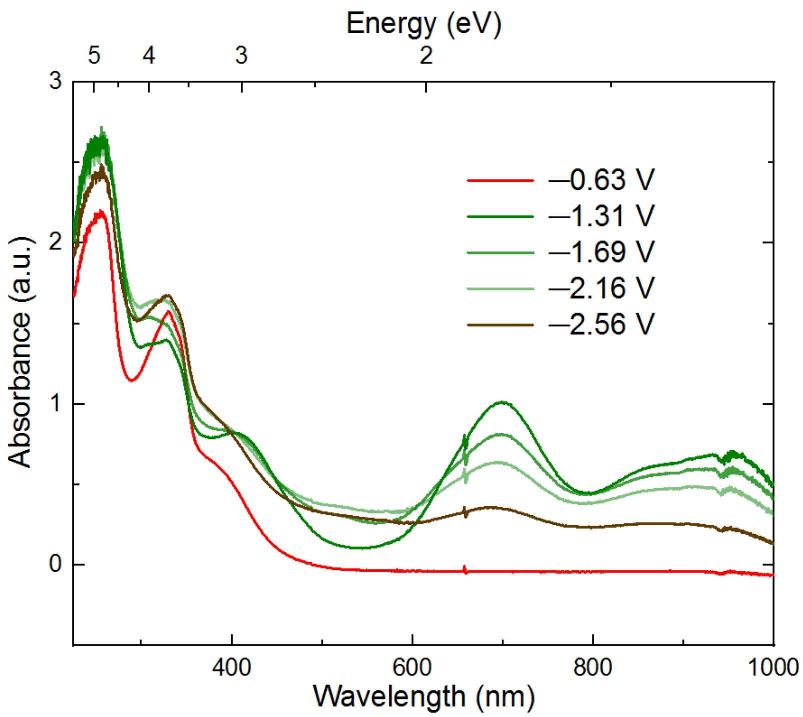

Figure S27. Electronic absorption spectra of the five accessible oxidation states of **3**.

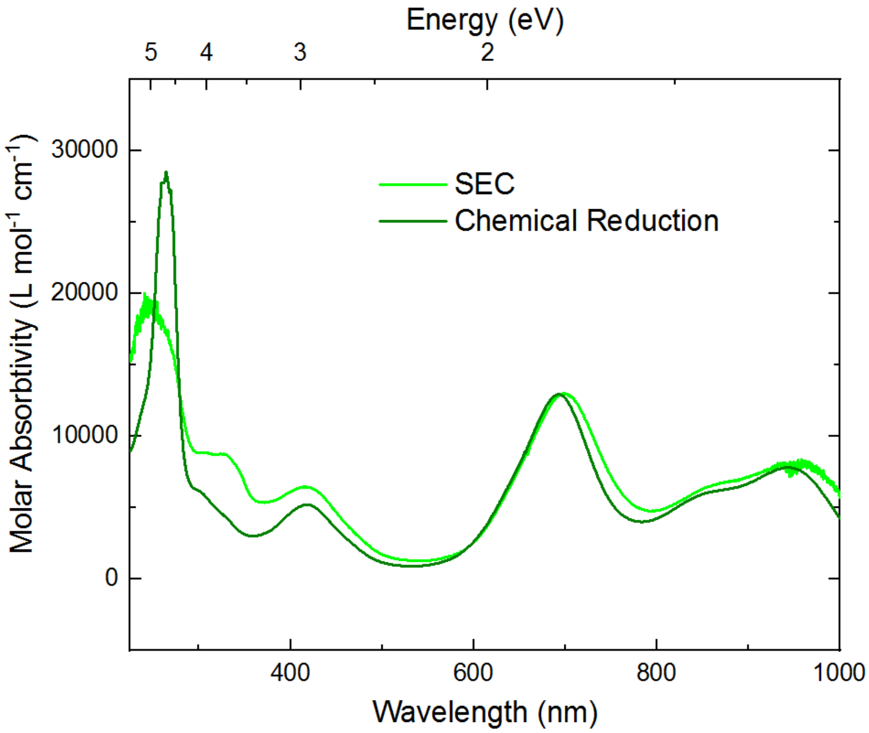

Figure S28. Electronic absorption spectra (THF) of electrochemically singly reduced **3** (produced by SEC) and **4** (produced by chemical reduction and isolation). SEC spectrum taken in the presence of 0.1 M [$^{n}Bu_4N$][PF_6] as electrolyte. Molar absorptivity calculated assuming complete conversion after constant current achieved in chronoamerometry experiment.

Crystallographic Information

Refinement Details for **3.**

A crystal of **3** was mounted on a nylon loop using Paratone oil under a nitrogen stream. Low temperature (200 K) X-ray data were obtained with a Bruker MicroStar microfocus rotating anode generator running at 50 mA and 45 kV (Cu Kα = 1.54178 Å; Apex II detector positioned at 50.0 mm and equipped with Helios multilayer mirror optics). All diffractometer manipulations, including data collection, integration and scaling were carried out using the Bruker APEXII software.[1] Absorption corrections were applied using SADABS.[2] The space group was determined on the basis of systematic absences and intensity statistics and the structure were solved were solved by intrinsic phasing using XT.[3] All non-hydrogen atoms were refined using anisotropic displacement parameters. Hydrogen atoms were placed in idealized positions and refined using a riding model. The structure was refined (weighted least squares refinement on F^2) to convergence using the Olex software package equipped with XL.[4]

Table S29: Crystal and Refinement Data

Compound	**3**
CCDC #	1842459
empirical formula	$C_{22}H_{24}ClF_6N_5O_4PRh$
formula wt	705.79
T (K)	199.99
a, Å	12.1943(2)
b, Å	18.7645(4)
c, Å	13.4798(2)
α, deg	90
β, deg	115.139(1)
γ, deg	90
V, $Å^3$	2792.29(9)
Z	4
cryst. syst	monoclinic
space group	$P2_{1/c}$
ρ_{calcd}, g/cm^3	1.679
2θ range, deg	8.008 to 140.59
μ, mm^{-1}	7.088
abs corr	Multi-scan
GOOF[c]	1.096
R1,[a] wR2[b] (I > 2σ(I))	0.0234, 0.0591

[a] $R1 = \Sigma||F_o|-|F_c|| / \Sigma|F_o|$ [b] $wR2 = [\Sigma [w(F_o^2-F_c^2)^2] / \Sigma [w(F_o^2)^2]]^{1/2}$ [c] $GOOF = S = [\Sigma [w(F_o^2-F_c^2)^2] / (n-p)]^{1/2}$

Special Refinement Details for 3.

In refinement of the structure of **3**, a single low-angle reflection (*hkl*: -2, 0, 2) was omitted due to abnormal intensity. The proximity of this reflection to the beamstop is likely responsible for the anomalous intensity. The structure of **3** contains a co-crystallized acetonitrile molecule.

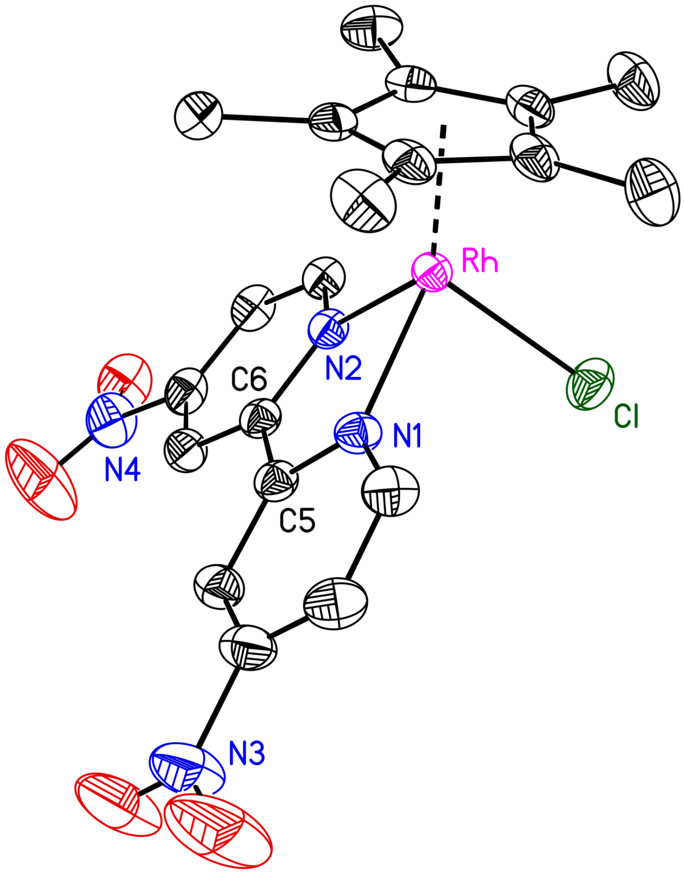

Figure S30: Solid-state structure of **3**. Hydrogen atoms, solvent, and counteranion are omitted for clarity. Displacement ellipsoids shown at the 50% probability level.

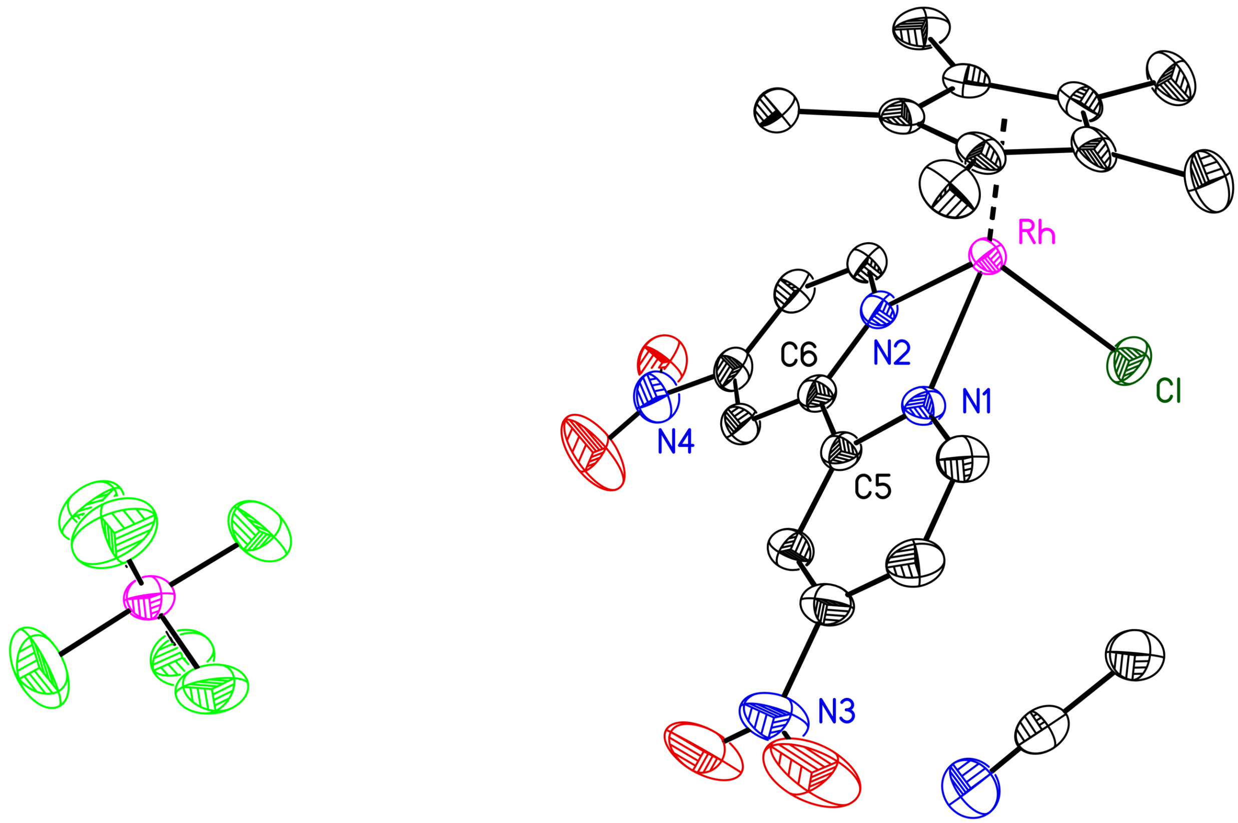

Figure S31: Full solid-state structure of **3**. Hydrogen atoms omitted for clarity. Displacement ellipsoids shown at the 50% probability level.

References

[1] *APEX2, Version 2 User Manual, M86-E01078,*; Bruker Analytical X-ray Systems: Madison, WI, June 2006.

[2] Sheldrick, G. M., SADABS (version 2008/1): Program for Absorption Correction for Data from Area Detector Frames, University of Göttingen, 2008

[3] Sheldrick, G. *Acta Crystallogr., Sect. A: Found. Crystallogr.* **2015**, *71*, 3-8.

[4] Dolomanov, O. V.; Bourhis, L. J.; Gildea, R. J.; Howard, J. A. K.; Puschmann, H. *J. Appl. Crystallogr.* **2009**, *42*, 339-341.
